# Accurate ^57^Fe Mössbauer Parameters from General Gaussian Basis Sets

**DOI:** 10.1021/acs.jctc.1c00722

**Published:** 2021-11-22

**Authors:** Gerard Comas-Vilà, Pedro Salvador

**Affiliations:** Institute of Computational Chemistry and Catalysis, Chemistry Department, University of Girona, Montilivi Campus, Girona, Catalonia 17003, Spain

## Abstract

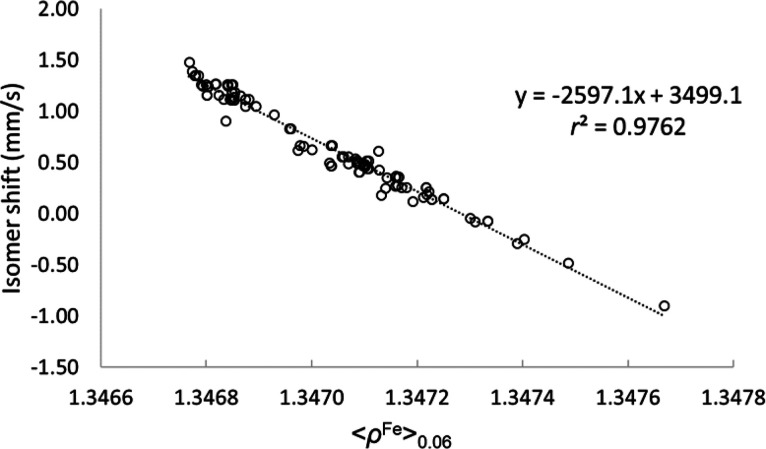

The prediction of isomer shifts in ^57^Fe Mossbauer spectra is typically achieved by building calibration lines using the values of the density at the nuclear position. Using Slater-type orbital basis or large and specific Gaussian-type orbital basis has been thus far mandatory to achieve accurate predictions with density functional theory methods. In this work, we show that replacing the value of the density at the nucleus by the density integrated in a sphere of radius 0.06 au centered on the Fe nuclei yields excellent calibration lines (*r*^2^ = 0.976) with a high predictive power (*q*^2^ = 0.975, MAE = 0.055 mm·s^–1^) while using the conventional def2-TZVP basis set and X-ray geometrical parameters. Our data set comprises 69 ^57^Fe-containing compounds and 103 signals. We also find B3LYP performing significantly better than the PW91 functional.

## Introduction

Since the discovery of the recoilless nuclear resonance fluorescence by Rudolf Mössbauer in 1958,^1^ Mössbauer spectroscopy has become a very important experimental technique, especially used for studying transition-metal compounds and metalloproteins, providing valuable information about their electronic and geometric characteristics.^2^ The most used element is by far the ^57^Fe nucleus as it is essential, very abundant in biological systems, and the quality of the signal is good.^3−6^

From the Mössbauer spectrum, one can extract two relevant parameters, namely the isomer shift (IS) and the quadrupole splitting (QS). The QS of an ^57^Fe nucleus for the nuclear excited state (*I* = 3/2) is expressed as
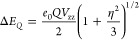
1where *e*_0_ is the electron charge, *Q* is the quadrupole moment of the ^57^Fe nucleus (the value of 0.16 barn determined by Dufek *et al.*(7) is typically used), and the asymmetry parameter η is defined as
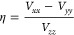
2

*V*_*xx*_, *V*_*yy*_, and *V*_*zz*_ are the eigenvalues of the electric field gradient at the nucleus, where |*V*_*zz*_| ≥ |*V*_*xx*_| ≥ |*V*_*yy*_|. QS can nowadays be directly evaluated with most codes, using electronic structure methods such as Kohn–Sham density functional theory (KS-DFT).

On the other hand, the IS originates from the energy difference between the γ-transitions in the source (*E*_s_) and the absorber (*E*_a_) nuclei^8^
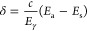
3where *c* is the velocity of light and *E*_γ_ is the energy of the incident γ photon. IS is commonly measured in terms of the Doppler velocity (mm/s) necessary for resonance. The IS can be essentially expressed in terms of electron density at the nucleus for the absorber, ρ_a_(0), and the source, ρ_s_(0), and the change in the nuclear radius *R* is^9^
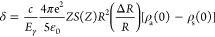
4where *Z* is the nuclear charge and *S*(*Z*) is a scaling factor correcting for relativistic effects. With few exceptions, as the same source is used for all the Mössbauer spectra, the IS for ^57^Fe species is most typically expressed in the literature as
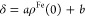
5where ρ^Fe^(0) is the calculated electron density at the nucleus, and coefficients *a* and *b* are empirically determined by a least-squares fit to experimental data.

This strategy has been widely used in order to estimate the IS of a wide range of ^57^Fe complexes.^8−14,16−18,20,21,23,24^ In most cases, KS-DFT methods have been used to determine the electron density, thus permitting to tackle rather large mono- and dinuclear iron-containing complexes with reasonable success but still exhibiting some limitations.

The nature of the underlying one-electron basis set in the KS-DFT calculations has been a major concern. Some of the first works of Noodleman and co-workers utilized Slater-type atomic orbitals (STOs).^10−12^ They used the PW91 functional in combination with the STO–TZP basis to fit eq 5 using a large training set of Fe complexes. However, when the fit was applied to Fe-containing active sites of several proteins, the predicted IS values were significantly larger than the experimental data. Hopmann *et al.*(13) also reported IS and QS calculations for 21 nonheme iron complexes combining the OLYP functional and the STO-TZP basis set.

Most of the studies with Gaussian-type orbital (GTO) basis sets make use of specific basis sets for the Fe center. Zhang *et al.*(14) compared the performance of pure and hybrid exchange–correlation functionals for a series of 24 Fe-containing systems. They explored the basis set dependence and found mandatory using a locally dense GTO basis for the Fe center.^15^ Neese^16^ also developed a specific core-polarized GTO basis set for first-row TM and used it to calibrate B3LYP and BP86 functionals for the prediction of IS using 15 iron compounds. Nemykin and Hadt^17^ showed a slightly better performance of B3LYP compared to BPW91 for a set of 36 compounds, employing Wachter’s full-electron basis set with one additional set of polarization functions for Fe. Interestingly, uncontracting the s-type basis functions did not lead to significant improvement of the IS values. Bochevarov *et al.*(18) investigated the prediction of IS and QS by eight functionals combined with two core-polarized Gaussian basis sets for 31 iron compounds. The fully uncontracted Partridge-1 basis set^19^ produced better linear fits of the IS values. In this study, different calibration lines were proposed according to different formal oxidation states (OS’s) of the Fe centers. This strategy had already been explored by Han *et al.*(20) to improve the linear fits at the PW91/def2-TZVP level of theory.

In 2013, Pápai *et al.*(21) reported one of the most extensive studies related with the calculation of Mössbauer parameters, using 66 iron compounds and comparing the STO and GTO basis sets. The authors ruled out the use of conventional GTO basis sets and turned to Neese’s CP(PPP) GTO basis set for the Fe centers. They obtained remarkably good calibration lines, independently of Fe’s formal OS.

Thus, most of the previous works using GTOs used large uncontracted or specific core-polarized basis sets, which even required adapting the numerical grid for the DFT calculations.^16^ An alternative to improve the linear fits is to produce different calibration lines for the different OS’s of the metal.^13,18,20^ This avenue is less desirable as the assignation of formal OS is not always unambiguous.^22^ As an exception, McWilliams *et al.*(23) reported IS calibration lines at the B3LYP/def2-TZVP level of theory, but both the training set (nine compounds) and the test set (n = 25) consisted solely of low-valent iron diketiminate complexes.

The aim of this work is to show that one can obtain high-quality, OS-independent, calibration lines for the IS from conventional (triple-zeta quality) GTO basis sets and for a large and chemically diverse set. We consider all X-ray structures of monomeric and dimeric Fe compounds gathered by Han *et al.*,^20^ Bochevarov *et al.*,^18^ and Sandala *et al.*(24) In addition, we also include the high-valent compounds reported in refs (25) and (26) (no X-ray available for the latter) and FeO_4_^2–^ ion, for a total of 69 iron-containing compounds and 103 signals, covering a wide range of formal oxidation states (+2, +2.5, +3, +3.5, +4, +6) and spin states. The experimental IS values range from −0.90 to +1.48 mm·s^–1^. The data set is described in Tables 1–3. It comprises (i) simple ions (n = 11), (ii) nitrosyl complexes (n = 6), (iii) Fe–S compounds (n = 8), (iv) porphyrin derivatives (n = 7), (v) (μ-oxo)di-iron (Fe–O–Fe), and (μ-hydroxo)di-iron (Fe–OH–Fe) units from dinuclear nonheme iron proteins (n = 17), (vi) di-iron with carboxylates as ligands present in bacterial multicomponent mono-oxygenases (n = 12), and (vii) iron with nitrogen, carbon, or oxygen derivative ligands (n = 8). To the best of our knowledge, the present work comprises the largest and most diverse data set in Mössbauer studies thus far.

**Table 1:** Calculated and Experimental IS’s (in mm/s) for (μ-oxo) and (μ-hydroxo) Di-iron Systems **1**–**17** and Some Mono-iron Complexes **18–25** Using ⟨ρ⟩_0.06_ Values at B3LYP/def2-TZVP Level of Theory^a^^,^^b^

			experimental	calculated			
complex	*S*_total_	OS	δ_4.2K_	ρ(0)	⟨ρ⟩_0.06_	δ	code
(1) Fe_2_(salmp)_2_^2–^	0	+2	1.11	11580.197	1.346847	1.13	KASFUF
		+2	1.11	11580.237	1.346853	1.12	
(1a) Fe_2_(salmp)_2_^2–^	4	+2	1.11	11580.194	1.346847	1.13	KASFUF
		+2	1.11	11580.234	1.346853	1.12	
(2) Fe_2_(OH)(OAc)_2_(Me_3_TACN)_2_^+^	0	+2	1.16	11580.006	1.346800	1.26	DIBWUG10
		+2	1.16	11580.010	1.346801	1.26	
(3) Fe_2_(salmp)_2_^–^	9/2	+2.5	0.83	11580.963	1.346960	0.84	KASGAM
		+2.5	0.83	11580.950	1.346958	0.85	
(4) Cl_3_FeOFeCl_3_^2–^	0	+3	0.36	11583.163	1.347164	0.31	FACTEI
		+3	0.36	11583.162	1.347164	0.31	
(5) Fe_2_O(OAc)_2_(Me_3_TACN)_2_^2+^	0	+3	0.47	11581.684	1.347037	0.64	DIBXAN10
		+3	0.47	11581.678	1.347036	0.65	
(6) Fe_2_O(OAc)_2_(bipy)_2_Cl_2_	0	+3	0.41	11582.077	1.347090	0.51	VABMUG
		+3	0.41	11582.091	1.347090	0.50	
(7) Fe_2_(salmp)_2_	0	+3	0.56	11581.633	1.347060	0.58	KASFOZ
		+3	0.56	11581.635	1.347060	0.58	
(8) Fe_2_(cat)_4_(H_2_O)_2_^2–^	0	+3	0.56	11581.811	1.347057	0.59	TEMKUR
		+3	0.56	11581.813	1.347057	0.59	
(9) Fe_2_(O)_2_(6-Me_3_-TPA)_2_^2+^	0	+3	0.50	11581.973	1.347086	0.52	YOCKAC
		+3	0.50	11581.973	1.347086	0.52	
(10) (Fe(Me_3_TACN)(TTC))_2_O	0	+3	0.46	11582.123	1.347100	0.48	YOHMOX
		+3	0.46	11582.123	1.347100	0.48	
(11) Fe_2_O_2_(5-Et_3_-TPA)_2_^3+^	9/2	+3.5	0.14	11582.786	1.347227	0.15	DEKNOW
		+3.5	0.14	11582.786	1.347227	0.15	
(12) (Fe(TAML)_2_)_2_O^2–^	0	+4	–0.07	11583.843	1.347333	–0.13	KAJBIH
		+4	–0.07	11583.843	1.347333	–0.13	
(13) Fe_2_(OH)(O_2_P(OPh)_2_)_3_(HBpz_3_)_2_^2+^	1	+3	0.44	11581.911	1.347108	0.46	PIMTAG
		+3	0.44	11581.896	1.347105	0.47	
(14) Fe_2_O(Piv)_2_(Me_3_TACN)_2_^2+^	0	+3	0.48	11582.036	1.347099	0.48	ZOCPEM
		+3	0.48	11582.058	1.347101	0.48	
(15) Fe_2_O(TMIP)_2_(OAc)_2_^2+^	0	+3	0.52	11582.000	1.347104	0.47	JIGNUI
		+3	0.52	11582.015	1.347108	0.46	
(16) Fe_2_O(HBpz_3_)_2_(OAc)_2_	0	+3	0.52	11581.856	1.347084	0.52	CACZIP10
		+3	0.52	11581.861	1.347086	0.51	
(17) Fe_2_OH(HBpz_3_)_2_(OAc)_2_	0	+3	0.47	11581.871	1.347100	0.47	COCJIN
		+3	0.47	11581.828	1.347097	0.48	
(18) Fe(phen)_2_Cl_2_	2	+2	1.05	11580.533	1.346874	1.06	CPENFE01
(19) Fe(opda)_2_Cl_2_	2	+2	0.91	11580.424	1.346837	1.17	FUJQOQ
(20) Fe(Py)_4_Cl_2_	2	+2	1.16	11580.219	1.346824	1.20	TPYFEC
(21) Fe(HB(mtda^R^)_3_)_2_	0	+2	0.49	11581.593	1.347070	0.56	JOHCEP
(22) [(Me_3_cy-ac)FeN]^2+^	0	+2	–0.29	11584.129	1.347389	–0.27	ref (26)
(23) FeCl(MBTHx)_2_	5/2	+3	0.43	11582.669	1.347128	0.40	CELVEU
(24) H_2_B(MesIm)_2_Fe(NMes)_2_	3/2	+3	–0.25	11584.484	1.347403	–0.31	ZACWUZ
(25) [H_2_B(MesIm)_2_Fe(NMes)_2_]^+^	0	+4	–0.48	11585.100	1.347486	–0.53	ZACXAG

a Electron densities in atomic units.

b The ligands are encoded as follows: salmp = 2-bis(salicylideneamino)methylphenolate, opda = 1,2-phenylenediamine, Me_3_TACN = 1,4,7-trimethyl-1,4,7-triazacyclonane, HB(mtda^R^)_3_ = tris(mercaptothiadiazolyl)borate, TPA = tris(2-pyridylmethyl)amine, cy-ac = anion of 1,4,8,11-tetraazacyclotetradecane-1-acetate, cat = catecholato-O,O,O′-*bis*(catecholato-O,O′), HBpz_3_ = hydrotis-1-(pyrazolyl)borate, Piv = pivalate, TTC = tetrachlorocatecholato-O,O′ dianion, TMIP = tris(methylimidazol-2-yl)phosphine, MBTHx = *bis*(*N*-methylbenzothiohydroxamato), H_2_B(MesIm)_2_ = dihydrobis[1-(2,4,6-trimethylphenyl)imidazole-2-ylidene]borato, and TAML = tetra-amido macrocyclic ligand.

**Table 2:** Calculated and Experimental IS’s (in mm/s) for Carboxylate Di-iron Complexes **26–37** and Nitrosyl Complexes **38–43** Using ⟨ρ⟩0.06 Values at B3LYP/def2-TZVP Level of Theory^a^^,^^b^

			experimental	calculated			
complex	*S*_total_	OS	δ_4.2K_	ρ(0)	⟨ρ⟩_0.06_	δ	code
(26) Fe_2_(μ–O_2_C–CH_3_)_4_(C_5_H_5_N)_2_	0	+2	1.12	11580.389	1.346848	1.13	EGAFUN
		+2	1.12	11580.358	1.346845	1.14	
(27) Fe_2_(μ–O_2_C–CH_3_)_2_(O_2_C–CH_3_)_2_–(THF)_2_	4	+2	1.26	11580.472	1.346851	1.12	EGAFAT
		+2	1.26	11580.428	1.346847	1.13	
(27a) Fe_2_(μ–O_2_C–CH_3_)_2_(O_2_C–CH_3_)_2_–(THF)_2_	0	+2	1.26	11580.378	1.346840	1.15	EGAFAT
		+2	1.26	11580.344	1.346839	1.15	
(28) Fe_2_(μ–O_2_C–CH_3_)_2_(O_2_C–CH_3_)_2_–(NH_2_CH_2_CH_3_)_2_	4	+2	1.19	11580.525	1.346848	1.13	ADIGID
		+2	1.19	11580.525	1.346849	1.13	
(28a) Fe_2_(μ–O_2_C–CH_3_)_2_(O_2_C–CH_3_)_2_–(NH_2_CH_2_CH_3_)_2_	0	+2	1.19	11580.531	1.346848	1.13	ADIGID
		+2	1.19	11580.547	1.346854	1.12	
(29) Fe_2_(μ–OH_2_)_2_(μ–O_2_C–CH_3_)_2_–(O_2_C–CH_3_)_3_(THF)_2_(OH_2_)	4	+2	1.35	11579.864	1.346780	1.31	FEMTEX
		+2	1.35	11579.769	1.346778	1.31	
(29a) Fe_2_(μ–OH_2_)_2_(μ–O_2_C–CH_3_)_2_–(O_2_C–CH_3_)_3_(THF)_2_(OH_2_)	0	+2	1.35	11579.919	1.346784	1.30	FEMTEX
		+2	1.35	11579.766	1.346778	1.31	
(30) Fe_2_BPMP(OPr)_2_^+^	0	+2	1.24	11579.595	1.346802	1.25	GATFUC
		+2	1.24	11579.599	1.346803	1.25	
(31) Fe(II)Fe(III)BPMP(OPr)_2_^2+^	1/2	+2	1.15	11580.316	1.346865	1.09	GATFOW
		+3	0.50	11581.514	1.347034	0.65	
(32) Fe_2_(O_2_CH)_2_(BIPhMe)_2_	0	+2	1.26	11579.831	1.346800	1.26	SISKOU
		+2	1.25	11580.319	1.346840	1.15	
(33) Fe_2_(OAc)_2_(TPA)_2_^2+^	0	+2	1.12	11580.138	1.346832	1.17	VUNMIA
		+2	1.12	11580.215	1.346844	1.14	
(34) Fe_2_(ImH)_2_(XDK)(O_2_CPh)_2_(MeOH)	0	+2	1.35	11579.625	1.346778	1.31	YUZKAF10
		+2	1.12	11580.718	1.346874	1.06	
(35) Fe_2_(py)_2_(O_2_CAr^Mes^)_4_	0	+2	1.14	11580.408	1.346853	1.12	XIGDIA
		+2	1.14	11580.409	1.346853	1.12	
(36) Fe_2_(H_2_O)(O_2_CPh)_4_(TMEN)_2_	0	+2	1.25	11579.860	1.346793	1.28	VUPJUL
		+2	1.26	11579.807	1.346791	1.28	
(37) Fe_2_(H_2_O)(OAc)_4_(TMEN)_2_	2	+2	1.27	11580.012	1.346818	1.21	VUPJOF
		+2	1.27	11580.013	1.346817	1.21	
(38) Fe(NO)_2_(S(p-Me)Ph)_2_^–^	2	+2	0.18	11582.701	1.347132	0.40	SONMUE
(39) [Fe(SC_2_H_3_N_3_)(SC_2_H_2_N_3_)(NO)_2_]	5/2	+3	0.19	11583.189	1.347218	0.17	EYABOV
(40) Fe_2_(S-*t*-Bu)_2_(NO)_2_	0	+3	0.15	11583.320	1.347249	0.09	GIDKIN02
		+3	0.15	11583.320	1.347249	0.09	
(41) Fe(S-*t*-Bu)_3_NO	5/2	+3	0.26	11583.138	1.347180	0.27	WEDXAF
(42) [Fe(NO)(dtc*i*-Pr_2_)_2_]	3/2	+3	0.35	11582.603	1.347143	0.37	PRCBFE
(43) [Fe_2_(NO)_2_(Et-HPTB)(O_2_CPh)]^2+^	0	+3	0.67	11581.513	1.347036	0.64	RABHAD
		+3	0.67	11581.532	1.347039	0.64	

a Electron densities in atomic units.

b The ligands are encoded as follows: BPMP = 2,6-*bis*(*bis*(2-pyridylmethyl) aminomethyl))-4-methylphenolato, BIPhMe = *bis*(1-methylamidazol-2-yl)phenylmethoxymethane, ImH = imidazole, XDK = acid anion of *m*-xylenediamine bis(Kemp’s triacid)-imide, HO_2_CAr^Mes^ = 2,6-*bis*(mesityl)benzoic acid, TMEN = N,N,N′,N′-tetramethylethylenediamine, Et-HPTB = *N*,*N*,*N*′,*N*′-tetrakis(*N*-ethyl-2-benzimidazolylmethyl)-1,3,diaminopropane).

**Table 3:** Calculated and Experimental IS’s (in mm/s) for Simple Fe Ions **44–54**, Fe–S Compounds **55–62,** and Fe-Porphyrin systems **63–69** Using ⟨ρ⟩0.06 Values at B3LYP/def2-TZVP Level of Theory^a^^,^^b^

			experimental	calculated			
Complex	*S*_total_	OS	δ_4.2K_	ρ(0)	⟨ρ⟩_0.06_	δ	code
(44) FeF_6_^4–^	2	+2	1.48	11579.703	1.346767	1.34	ICSD 26603
(45) FeCl_4_^2–^	2	+2	1.05	11581.197	1.346893	1.01	DEBWEM
(46) FeBr_4_^2–^	2	+2	1.12	11581.194	1.346881	1.05	DEBWIQ
(47) Fe(NCS)_4_^2–^	2	+2	0.97	11581.038	1.346928	0.92	KEFFEG
(48) Fe(H_2_O)_6_^2+^	2	+2	1.39	11579.688	1.346772	1.33	ICSD 16589
(49) Fe(bipy)_2_Cl_2_^+^	5/2	+3	0.54	11582.032	1.347082	0.52	CAVDOS05
(50) FeF_6_^3–^	5/2	+3	0.61	11582.155	1.347126	0.41	TUKBOQ
(51) FeCl_6_^3–^	5/2	+3	0.56	11582.177	1.347069	0.56	DALLIL
(52) FeCl_4_^–^	5/2	+3	0.36	11583.208	1.347158	0.33	MICYFE10
(53) FeO_4_^2–^	1	+6	–0.90	11587.208	1.347668	–1.01	ICSD 32756
(54) FeCl_5_(H_2_O)^2–^	5/2	+3	0.49	11582.272	1.347090	0.50	VOCBAQ
(55) Fe(DTSQ)_2_^2–^	2	+2	0.67	11582.068	1.346977	0.80	PTSQFE10
(56) Fe(SPh)_4_^2–^	2	+2	0.66	11581.983	1.346984	0.78	PTHPFE10
(57) [Fe_2_S_2_(S_2_-*o*-xyl)_2_]^2–^	5	+3	0.28	11583.315	1.347162	0.32	XLDTSF
		+3	0.28	11583.315	1.347162	0.32	
(58) [Fe_2_S_2_(OPh-*p*-CH_3_)_4_]^2–^	5	+3	0.37	11583.059	1.347159	0.32	GIBCUP
		+3	0.37	11583.058	1.347159	0.32	
(59) [Fe_2_S_2_(C_4_H_4_N)_4_]^2–^	5	+3	0.26	11582.859	1.347217	0.17	CONSED10
		+3	0.26	11582.860	1.347217	0.17	
(60) Fe(SEt)_4_^–^	5/2	+3	0.25	11583.239	1.347139	0.37	CANDAW10
(61) Fe(PPh_3_)_2_(“S2”)_2_	1	+4	0.16	11583.052	1.347211	0.19	SOCVUB
(62) Fe(PPh_3_)(“ S2”)_2_	0	+4	0.12	11583.183	1.347192	0.24	SOCWAI
(63) Fe(OEP)CO	0	+2	0.27	11582.661	1.347158	0.33	YEQPOA
(64) Fe(OEP)	1	+2	0.63	11581.467	1.346999	0.74	DEDWUE
(65) Fe(OEC)	1	+2	0.62	11580.971	1.346973	0.81	BUYKUB10
(66) Fe(OEC)Cl	3/2	+3	0.22	11583.057	1.347222	0.16	SUMWUS
(67) Fe(OEC)C_6_H_5_	3/2	+3	–0.08	11583.786	1.347310	–0.07	SUMXED
(68) FeCl(η^4^-MAC*)^−^	5/2	+3	–0.04	11583.666	1.347301	–0.04	JESGUJ
(69) Fe(OEP)(4-NMe_2_Py)_2_^2+^	1/2	+3	0.26	11582.271	1.347170	0.30	VOFLOR

a Electron densities in atomic units.

b The ligands are encoded as follows: OEC = dianion of *trans*-7,8-dihydro-octaethylporphyrin, OEP = dianion of octaethylporphyrin, DTSQ = *bis*(dithiodithiosquarato,S,S′), η^4^-MAC* = 13,13-diethyl-2,2,5,5,7,7,10,10-octamethyl-1,4,8,11-tetra-azatetradecan-3,6,9,12,14-pentaone-*N*,*N*′,*N*″,*N*‴, “S2” = 1,2-benzenedithiolato-S,S′ dianion.

There are both numerical and fundamental issues concerning the determination of the electron density at the nuclear position with finite basis sets. From a numerical perspective, the accuracy on the position of the nuclei is most relevant. Zhang *et al.* found significant deviations on the values of ρ^Fe^(0) depending on the number of digits of the Cartesian coordinates.^14^ Hence, the resolution of the X-ray structures, whether using X-ray *versus* optimized structures or the fact that the maximum of the density does not necessarily coincide with the exact nuclear position, also influences ρ^Fe^(0) values. On the other hand, it is well known that STOs can reproduce the cusp at the nuclei, while GTOs do not. Moreover, in the relativistic framework, the density diverges at the nuclear positions. To sort out this problem, the value of the density can be averaged over the surface of a sphere of finite radius, accounting for the nuclear volume, as implemented in ADF package.^27,28^ Sandala *et al.* recently compared the results obtained with this procedure and the usual density at the nucleus and found virtually no difference in the quality of the calibration lines.^24^ Yet, in this work, we explore the possibility of increasing the radius of the sphere well beyond the size of the nucleus (*ca.* 10^–14^ m) so that we in fact replace ρ^Fe^(0) in eq 5 by the density averaged over a sphere of radius *R*, ⟨ρ^Fe^⟩_R_.

## Computational Details

All calculations using PW91^29,30^ and B3LYP^31−33^ functionals in combination with the triple-zeta Gaussian-type basis set (def2-TZVP)^34^ were carried out with Gaussian09.^35^ The electron density integrated inside a sphere of radius *R* centered on the Fe atoms was implemented in our in-house program APOST-3D.^36^ For that purpose, a spherical grid^37^ of 30 radial and 110 angular points was used.

## Results and Discussion

**Figure 1:**
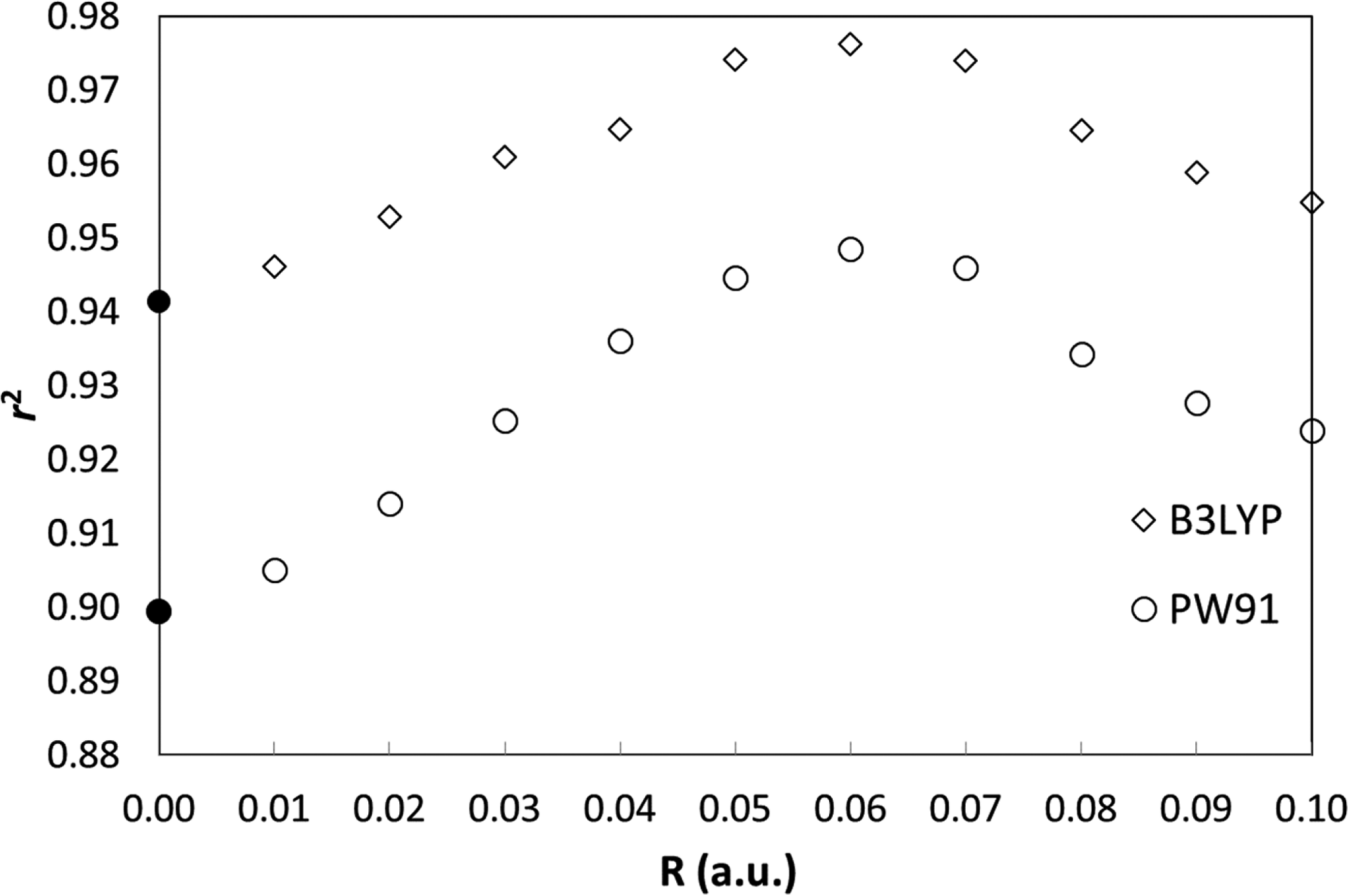
Square of the correlation coefficient of the IS calibration *vs* the radius of the sphere around Fe. Values at *R* = 0 correspond to the calibration lines calculated using ρ^Fe^(0).

We have performed single-point calculations on the X-ray geometries of our data set using both PW91 and B3LYP exchange–correlation functionals in combination with the conventional def2-TZVP basis set. In Figure 1 we depict the variation of the *r*^2^ values of the respective calibration lines for the IS with the radius of the sphere around the Fe atom.

**Figure 2:**
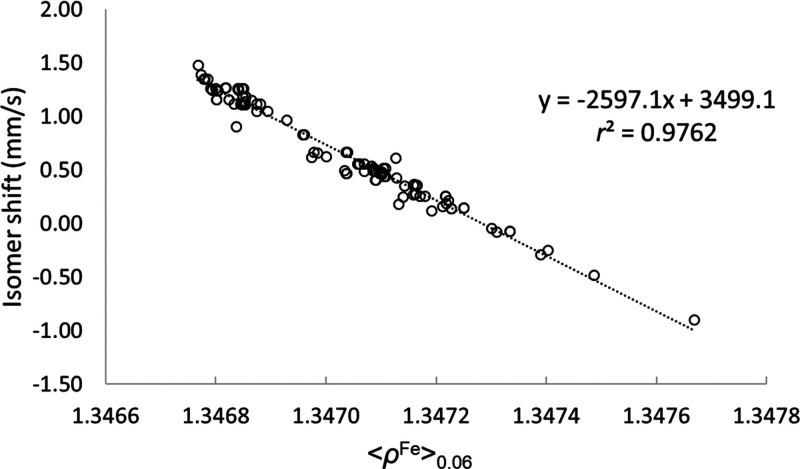
IS calibration line for the B3LYP/def2-TZVP level of theory using all Fe complexes (103 Fe sites in total).

It can be seen that replacing ρ^Fe^(0) by ⟨ρ^Fe^⟩_R_ leads to a *systematic increase* of the *r*^2^ values of the calibration lines for both functionals up until *ca. R* = 0.06 au. The calibration lines obtained using this optimal *R* value are significantly better than those obtained using the values of the density at the nuclear position, especially for the PW91 functional. Still, the best calibration line (shown in Figure 2) is obtained with the B3LYP functional.

Our *r*^2^ value is similar to the one reported by McWilliams *et al.*(23) also using conventional GTOs (0.974). However, our results were obtained on a much larger and more diverse test set.

**Figure 3:**
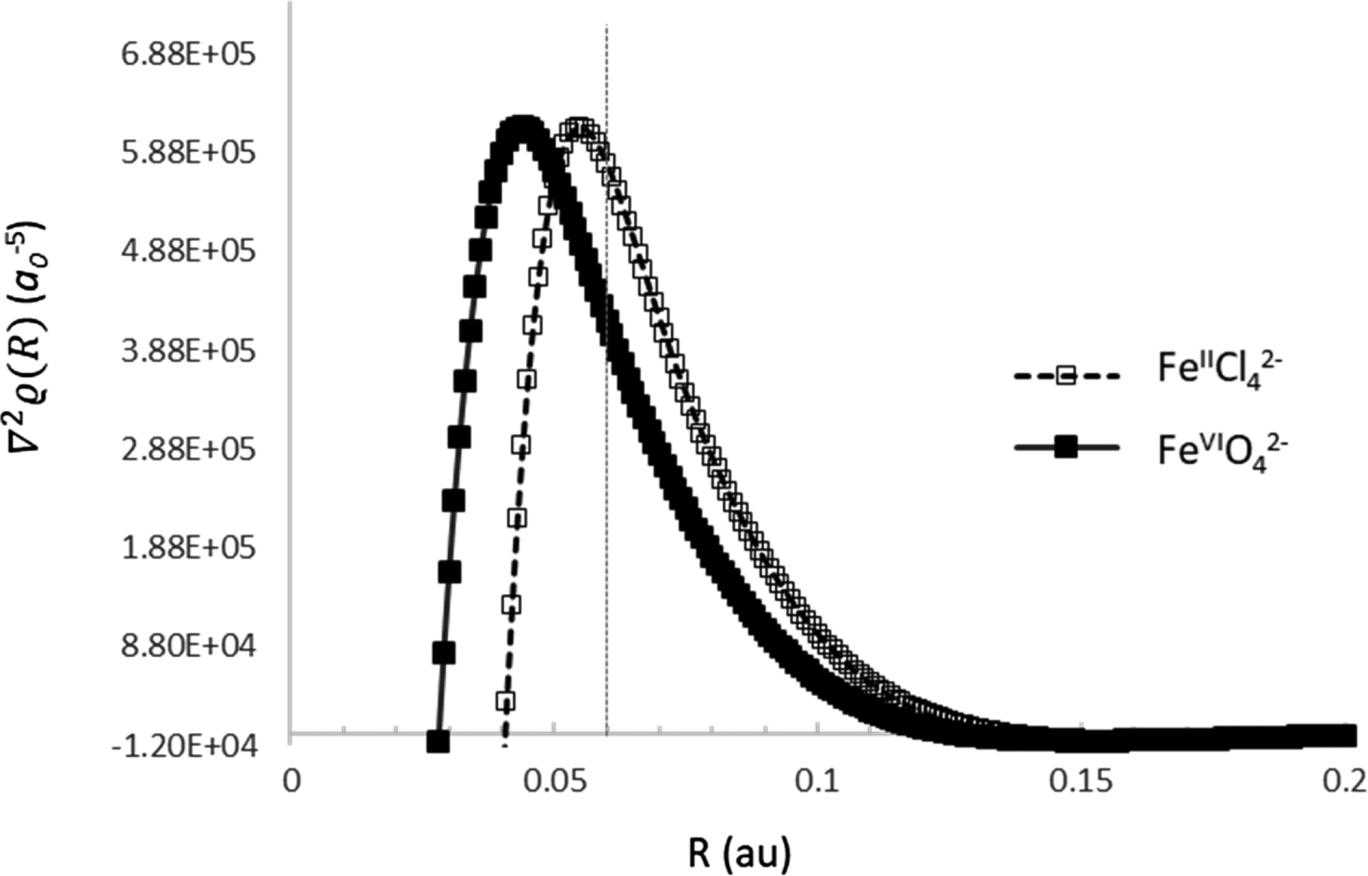
Values of the Laplacian of the density along the Fe–Cl and Fe–O bonds for FeCl_4_^2–^ and FeO_4_^2–^ ions, respectively.

The systematic improvement of the IS calibration lines (both in terms of *r*^2^ and RMSD of the predicted values) with the radius of the sphere up to a given optimal distance can be rationalized as follows. First of all, we should note that the partial atomic charges (*e.g.*, obtained with the TFVC method^38^) on the Fe center do not show any correlation at all with the experimental IS values (*r*^2^ = 0.02), even though the IS values are a pointer to the Fe oxidation state. We consider complexes in a wide range of oxidation states (from +2 to +6), but the Fe partial charges do not vary much (+0.92 to +1.97 for the whole set). Notice that partial charge is not a noninteger version of the OS, and both quantities often do not even correlate. Hence, as *R* increases, the integrated density tends toward a (rudimentary) atomic population measure, which is expected to perform poorly. In fact, when using *R* = 0.3 au, the *r*^2^ value already drops down to 0.47 and the RMSD increases up to 0.35 mm·s^–1^. Therefore, it is clear that the quality of the fit must decrease for large values of *R*. On the other hand, using solely the density at the nuclear position has a number of potential issues that have been discussed above. While some studies^24^ do not show significant improvement using the averaged density over the surface of a small sphere simulating the finite-size radius of the Fe nucleus (in combination with STOs), here it does help for conventional Gaussian-type basis sets that do not properly describe the density cusp at the Fe center. Hence, considering the average density within a small sphere (but not so small as to mimic the finite nucleus size) fixes some of these problems and increases the quality of the fit. What is remarkable from Figure 1 is that (i) the curve is smooth and (ii) the same optimal *R* value is obtained for the two DFT functionals used, also including implicit solvent effects (*vide infra*). In order to shed light into the particular optimum value of 0.06 au, we have depicted in Figure 3 the values of the Laplacian of the density (a well-known indicator of the shell structure^39,40^) around the Fe nucleus for the Fe^II^ and Fe^VI^ species, with distinct IS values. It can be readily seen that a maximum of the Laplacian indicating the charge depletion after the first (K) shell is found in both systems at a distance of *ca.* 0.05 au from the Fe center, very close to our optimal sphere radius value.

**Figure 4:**
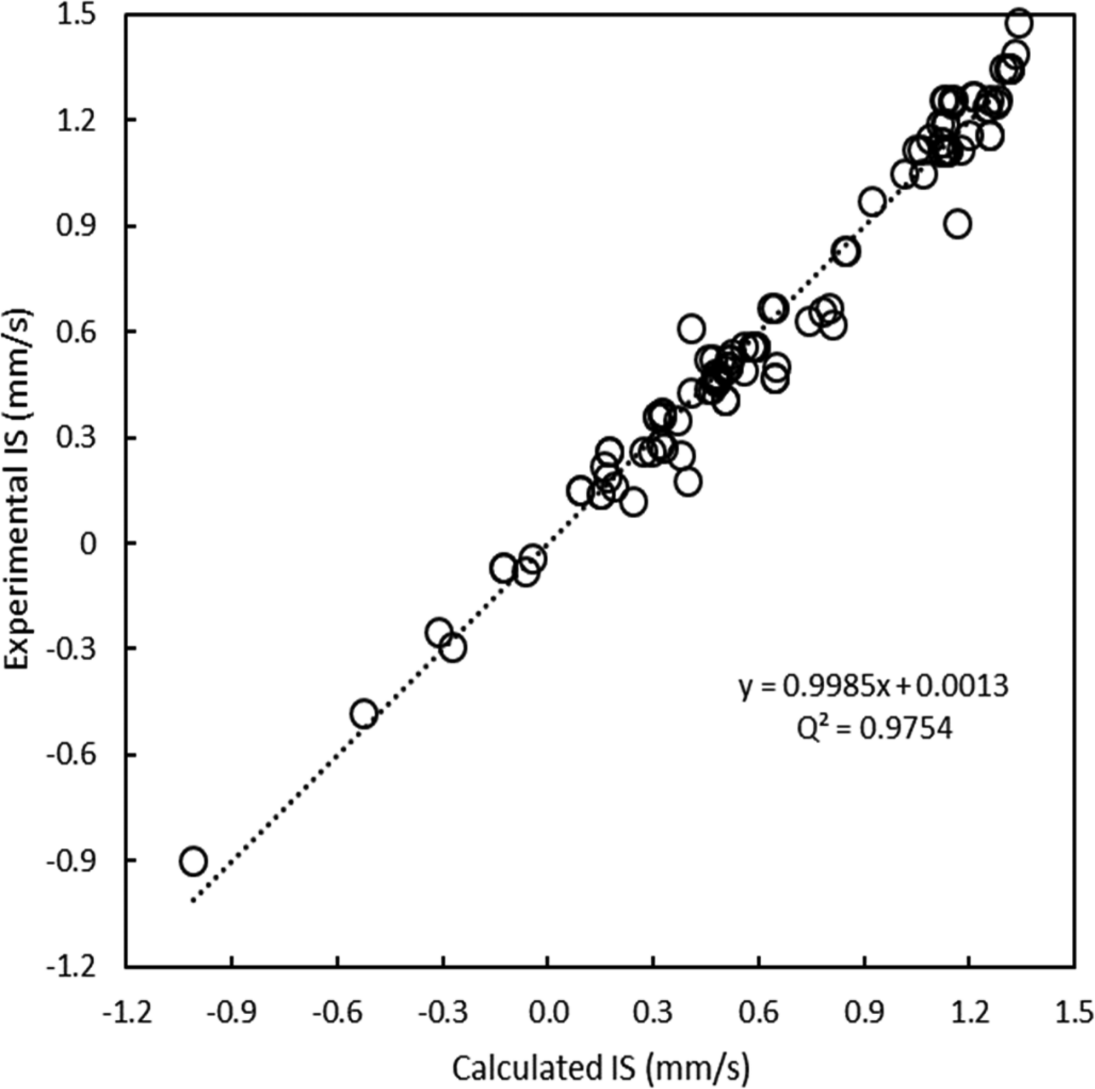
Cross-validation results for the calibration line of Figure 2.

On the other hand, a good model equation should also exhibit proper predictive power. For that reason, we have applied the leave-one-out cross-validation (LOOCV) strategy^41^ in which each data point is successively “left out” from the sample (*n*) and used for the validation and the remaining (*n* – 1) data samples. As shown in Figure 4, we obtain a cross-validation coefficient *q*^2^ = 0.975 and a mean absolute error (MAE) of the predicted IS values of 0.055 mm·s^–1^.

Let us discuss the quality of the predicted IS for each of the subsets. It is important to keep in mind that the predictions have been obtained with a unique calibration fit. For both the di-iron (Fe–O–Fe) and (Fe–OH–Fe) systems **1**–**17,** the predicted IS values are in excellent agreement with the experimental data. The calculated values are within 0.1 mm·s^–1^ of the experimental IS values, with the only exception of species **5** (0.47 mm·s^–1^, experimental, *vs* 0.65 mm·s^–1^, calculated). In the case of general mono-iron complexes, Fe(opda)_2_Cl_2_ (0.91 mm·s^–1^, experimental, *vs* 1.17 mm·s^–1^, calculated) also exhibits somewhat too large deviation. Remarkably, in the case of [(Me_3_cy-ac)FeN]^2+^ cation, the prediction is excellent, even though for this system we relied on a DFT-optimized structure.^26^

The results for the carboxylate di-iron complexes **26**–**37** (see Table 2) also exhibit very small deviations with respect to the experimental data. The worst case corresponds to the dinuclear mixed-valence *S* = 1/2 compound **31**. It originates from the antiferromagnetic coupling of high-spin Fe(II) and Fe(III) centers, which is probably rather challenging for a single-determinant KS-DFT description. A rather significant deviation (0.50 mm·s^–1^ (experimental) *vs* 0.65 mm·s^–1^ (calculated)) is found for the Fe(III) center.

The Fe-nitrosyl systems exhibit very small values of IS and have proven to be difficult for IS prediction.^18^ Our results are in very good agreement, with the only exception of species **38** (0.18 mm·s^–1^ (experimental) *vs* 0.40 mm·s^–1^ (calculated)).

The results obtained for the simple Fe ions **44**–**54** are gathered in Table 3. They involve highly charged species such as FeF_6_^4–^, with the largest IS of the set (1.48 mm·s^–1^), or the hexavalent ferrate anion, exhibiting a large and negative signal at −0.90 mm·s^–1^. The predicted IS values are rather good in both cases (1.34 mm·s^–1^ and −1.01 mm·s^–1^, respectively). Nevertheless, as all our calculations were performed in gas phase, we explored the effect of applying an implicit solvation model to the KS-DFT electron density calculations. We recomputed the electron densities for the full set using B3LYP+PCM (ε = 80) and rebuilt the universal calibration line. The results were rather discouraging. For the FeF_6_^3–^ anion, the predicted IS without solvent corrections is 0.41 mm·s^–1^. Including PCM, the value is 0.39 mm s^–1^, still somewhat off from the experimental reference (0.61 mm s^–1^). In the case of the FeO_4_^2–^ anion, including PCM also worsens the prediction (−1.01 mm s^–1^*vs* −1.13 mm s^–1^ with PCM; experimental value, −0.91 mm s^–1^). Some improvement was observed in some cases, like for species **65** (0.81 mm s^–1^*vs* 0.71 mm s^–1^ with PCM; experimental value, 0.62 mm s^–1^), but the overall quality of the fit decreases upon the inclusion of implicit solvation. Remarkably, the best B3LYP+PCM fit was once again observed for *R* = 0.06 au (see the Supporting Information).

**Figure 5:**
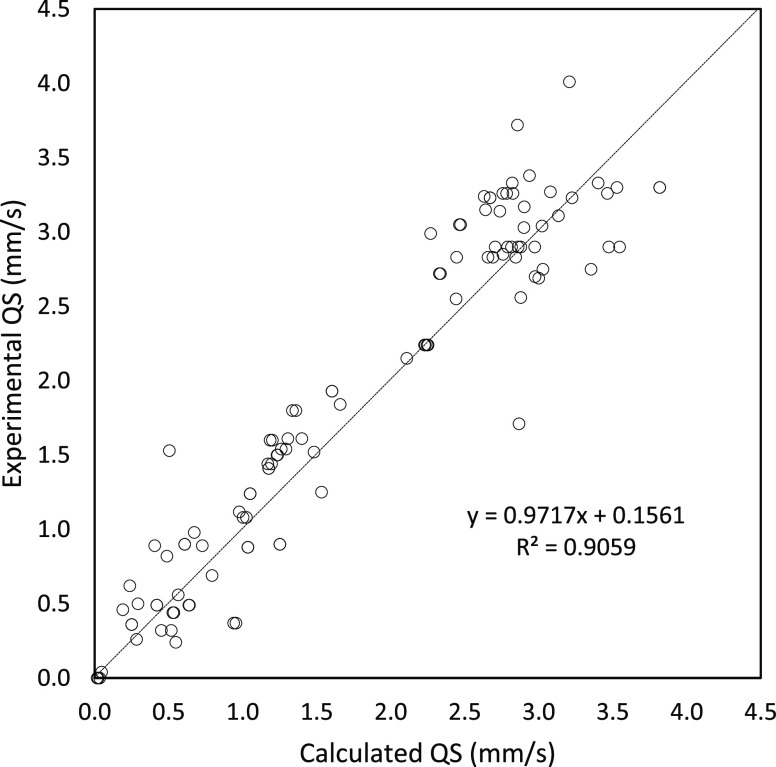
Correlation between the experimental and calculated QS (B3LYP) values for all Fe complexes (103 Fe sites in total).

The quality of the wave functions utilized for the IS predictions above can be independently proved by analyzing the performance on the calculation of the QS parameters. The calculated QS and η values are gathered in Tables S1 and S2 of the Supporting Information. Figure 5 depicts the comparison of the experimental and the B3LYP/def2-TZVP QS values. The *r*^2^ value (0.91) is somewhat smaller than the IS value at the same level of theory, but the associated MAE (0.28 mm·s^–1^) is much larger. This is rather expected as the QS parameter exhibits a range wider than the IS. The main outlier of the B3LYP results is the triplet Fe(II) porphyrin species Fe(OEP) **64** (1.71 mm·s^–1^ (experimental) *vs* 2.87 mm·s^–1^ (calculated)). Such discrepancy has already been analyzed in detail by Pápai^21^ and most recently by Gallenkamp *et al.*(42) and is connected with the existence of several low-lying electronic states for porphyrinic D_4h_ species. The discrepancy can be solved by introducing constraints to the wave function or switching to a GGA functional, as shown by Pápai.^21^ Indeed, our PW91 results yield a pretty accurate QS value for this compound (1.38 mm·s^–1^).

Bochevarov *et al.*(18) analyzed the performance of different functionals in combination with the core-polarized basis set and found the best result for O3LYP/Partridge-1, with a MAE of 0.28 mm·s^–1^ for a set of 31 systems (35 signals). McWilliams *et al.*(23) found a similar performance of def2-TZVP and CP(PPP) basis sets for a rather homogeneous set of 34 compounds and advocated the former due to its reduced computational cost. However, Pápai *et al.*(21) reported MAE values of 0.24 and 0.21 mm·s^–1^ using B3LYP in combination with CP(PPP) and STO-TZP basis, respectively, over their diverse set of 66 compounds. Including implicit solvation with COSMO worsened the results (MAE = 0.25 mm·s^–1^ in both cases). Thus, it appears that both core-polarized and STO-type basis sets tend to exhibit somewhat better performance than our B3LYP/def2-TZVP results.

On the other hand, PW91 performs consistently worse than B3LYP for our set, leading to *r*^2^ = 0.88 and MAE = 0.43 mm·s^–1^. The MAE value is significantly larger than that reported by previous studies. For instance, Liu *et al.*(12) reported a standard deviation of 0.30 mm·s^–1^ using PW91 in combination with a STO basis for a reduced set of 21 compounds. Later on, Han *et al.*(20) reported a MAE value of 0.25 mm·s^–1^ for a set of 35 compounds treated at the PW91/def2-TZVP level of theory. Contrary to Pápai’s finding, the authors found significant improvement by including implicit solvation with COSMO.

## Conclusions

DFT calculations have been carried out on the X-ray structures of a chemically diverse set of 69 ^57^Fe-containing compounds to calibrate the Mössbauer IS and QS parameters. We have explored for the first time the possibility of replacing the values of the density at the nucleus by the density integrated inside a sphere of variable radius centered on the Fe nuclei. The quality of the fit, quantified by the *r*^2^ values, increases monotonically until a radius of 0.06 au for both B3LYP and PW91 functionals. The predictive power of our universal calibration lines obtained with the general defTZVP basis set is comparable to that of previous studies using the STO basis, core-polarized or uncontracted GTOs. We also find B3LYP functional outperforming the PW91 functional for the prediction of both IS and QS parameters.

## Supporting Information Available

Experimental and calculated IS and QS for the whole dataset and calibration lines at B3LYP and PW91 levels of theory (PDF)
