# Supplementary material for: Accurate 57Fe Mössbauer Parameters from General Gaussian Basis Sets
Source: J Chem Theory Comput. 2021 Nov 22;17(12):7724–31. doi: 10.1021/acs.jctc.1c00722 (PMC8675134; doi:10.1021/acs.jctc.1c00722)
Supplement: Supplementary file 1 — ct1c00722_si_001.pdf [file ct1c00722_si_001.pdf]

Supporting Information

Accurate ^{57}Fe Mössbauer parameters from general Gaussian basis sets

Gerard Comas-Vilà and Pedro Salvador

Contents:

Tables S1 and S2

Figures S1-S7

Computational Details

References

Table S1: Calculated and Experimental Isomer Shifts and Quadrupole splitting (in mm/s) for the molecular set using $\langle\rho^{Fe}\rangle_{0.06}$ values at B3LYP/def2tzvp level of theory. Electron densities in atomic units.

			Experimental		Calculated							Code
Complex	S_{total}	OS	$\delta_{4.2K}$	ΔE_Q	$\rho(0)$	$\langle\rho\rangle_{0.06}$	δ_{calc}	V_{zz}, a.u.	$eQV_{zz}/2$	$\eta_{(calc)}$	$\Delta E_{Q(calc)}$	
(1) $Fe_2(salmp)_2^{2-}$	0	+2	1.11	2.24	11580.197	1.346847	1.13	1.37	2.21	0.23	2.23	KASFUF
		+2	1.11	2.24	11580.237	1.346853	1.12	1.37	2.22	0.22	2.23	
(1a) $Fe_2(salmp)_2^{2-}$	4	+2	1.11	2.24	11580.194	1.346847	1.13	1.37	2.22	0.29	2.25	KASFUF
		+2	1.11	2.24	11580.234	1.346853	1.12	1.38	2.22	0.28	2.25	
(2) $Fe_2(OH)(OAc)_2(Me_3TACN)_2^{+}$	0	+2	1.16	2.83	11580.006	1.346800	1.26	1.66	2.69	0.05	2.69	DIBWUG10
		+2	1.16	2.83	11580.010	1.346801	1.26	1.64	2.65	0.09	2.66	
(3) $Fe_2(salmp)_2^{-}$	9/2	+2.5	0.83	1.08	11580.963	1.346960	0.84	0.54	0.87	0.98	1.00	KASGAM
		+2.5	0.83	1.08	11580.950	1.346958	0.85	0.55	0.90	0.96	1.02	
(4) $Cl_3FeOFeCl_3^{2-}$	0	+3	0.36	1.24	11583.163	1.347164	0.31	0.65	1.05	0.04	1.05	FACTEI
		+3	0.36	1.24	11583.162	1.347164	0.31	0.65	1.05	0.06	1.05	
(5) $Fe_2O(OAc)_2(Me_3TACN)_2^{2+}$	0	+3	0.47	1.50	11581.684	1.347037	0.64	0.73	1.18	0.53	1.24	DIBXAN10
		+3	0.47	1.50	11581.678	1.347036	0.65	0.73	1.18	0.51	1.23	
(6) $Fe_2O(OAc)_2(bipy)_2Cl_2$	0	+3	0.41	1.80	11582.077	1.347090	0.51	0.82	1.33	0.14	1.34	VABMUG
		+3	0.41	1.80	11582.091	1.347090	0.50	0.84	1.35	0.16	1.36	
(7) $Fe_2(salmp)_2$	0	+3	0.56	0.88	11581.633	1.347060	0.58	0.61	0.98	0.56	1.03	KASFOZ
		+3	0.56	0.88	11581.635	1.347060	0.58	0.61	0.98	0.56	1.04	
(8) $Fe_2(cat)_4(H_2O)_2^{2-}$	0	+3	0.56	0.90	11581.811	1.347057	0.59	0.71	1.15	0.76	1.25	TEMKUR
		+3	0.56	0.90	11581.813	1.347057	0.59	0.71	1.15	0.76	1.25	
(9) $Fe_2(O)_2(6\text{-}Me_3\text{-}TPA)_2^{2+}$	0	+3	0.50	1.93	11581.973	1.347086	0.52	0.91	1.48	0.74	1.60	YOCKAC
		+3	0.50	1.93	11581.973	1.347086	0.52	0.91	1.48	0.74	1.60	
(10) $(Fe(Me_3TACN)(TTC))_2O$	0	+3	0.46	1.41	11582.123	1.347100	0.48	0.67	1.09	0.70	1.18	YOHMOX
		+3	0.46	1.41	11582.123	1.347100	0.48	0.67	1.09	0.70	1.18	
(11) $Fe_2O_2(5\text{-}Et_3\text{-}TPA)_2^{3+}$	9/2	+3.5	0.14	0.49	11582.786	1.347227	0.15	0.24	0.39	0.72	0.42	DEKNOW
		+3.5	0.14	0.49	11582.786	1.347227	0.15	0.24	0.39	0.72	0.42	
(12) $(Fe(TAML)_2)_2O^{2-}$	0	+4	-0.07	3.30	11583.843	1.347333	-0.13	2.14	3.47	0.80	3.82	KAJBIH
		+4	-0.07	3.30	11583.843	1.347333	-0.13	2.15	3.47	0.80	3.82	
(13) $Fe_2(OH)(O_2P(OPh)_2)_3(HBpz_3)_2^{2+}$	1	+3	0.44	0.44	11581.911	1.347108	0.46	0.33	0.53	0.26	0.53	PIMTAG
		+3	0.44	0.44	11581.896	1.347105	0.47	0.32	0.52	0.26	0.53	

cont.

(14) $Fe_2O(Piv)_2(Me_3TACN)_2^{2+}$	0	+3	0.48	1.54	11582.036	1.347099	0.48	0.77	1.24	0.30	1.26	ZOCPEM
		+3	0.48	1.54	11582.058	1.347101	0.48	0.78	1.27	0.35	1.29	
(15) $Fe_2O(TMIP)_2(OAc)_2^{2+}$	0	+3	0.52	1.61	11582.000	1.347104	0.47	0.86	1.39	0.20	1.40	JIGNUI
		+3	0.52	1.61	11582.015	1.347108	0.46	0.80	1.30	0.22	1.31	
(16) $Fe_2O(HBpz_3)_2(OAc)_2$	0	+3	0.52	1.60	11581.856	1.347084	0.52	0.64	1.03	0.98	1.19	CACZIP10
		+3	0.52	1.60	11581.861	1.347086	0.51	0.66	1.07	0.88	1.20	
(17) $Fe_2OH(HBpz_3)_2(OAc)_2$	0	+3	0.47	0.37	11581.871	1.347100	0.47	0.55	0.89	0.56	0.94	COCJIN
		+3	0.47	0.37	11581.828	1.347097	0.48	0.56	0.91	0.55	0.95	
(18) $Fe(phen)_2Cl_2$	2	+2	1.05	3.15	11580.533	1.346874	1.06	1.55	2.50	0.58	2.64	CPENFE01
(19) $Fe(opda)_2Cl_2$	2	+2	0.91	3.17	11580.424	1.346837	1.17	1.77	2.87	0.26	2.90	FUJQOQ
(20) $Fe(Py)_4Cl_2$	2	+2	1.16	3.14	11580.219	1.346824	1.20	1.69	2.74	0.01	2.74	TPYFEC
(21) $Fe(HB(mtda^R)_3)_2$	0	+2	0.49	0.26	11581.593	1.347070	0.56	0.15	0.25	0.97	0.28	JOHCEP
(22) $[(Me_3cy\text{-}ac)FeN]^{2+}$	0	+2	-0.29	1.53	11584.129	1.347389	-0.27	0.28	0.46	0.79	0.50	Ref 1
(23) $FeCl(MBTHx)_2$	5/2	+3	0.43	0.98	11582.669	1.347128	0.40	0.37	0.59	0.94	0.67	CELVEU
(24) $H_2B(MesIm)_2Fe(NMes)_2$	3/2	+3	-0.25	0.82	11584.484	1.347403	-0.31	0.29	0.47	0.49	0.49	ZACWUZ
(25) $[H_2B(MesIm)_2Fe(NMes)_2]^+$	0	+4	-0.48	1.25	11585.100	1.347486	-0.53	0.93	1.50	0.35	1.53	ZACXAG
(26) $Fe_2(\mu\text{-}O_2C\text{-}CH_3)_4(C_5H_5N)_2$	0	+2	1.12	3.05	11580.389	1.346848	1.13	1.45	2.34	0.57	2.46	EGAFUN
		+2	1.12	3.05	11580.358	1.346845	1.14	1.47	2.37	0.52	2.47	
(27) $Fe_2(\mu\text{-}O_2C\text{-}CH_3)_2(O_2C\text{-}CH_3)_2\text{-}(THF)_2$	4	+2	1.26	2.90	11580.472	1.346851	1.12	1.73	2.80	0.16	2.82	EGAFAT
		+2	1.26	2.90	11580.428	1.346847	1.13	1.71	2.76	0.24	2.79	
(27a) $Fe_2(\mu\text{-}O_2C\text{-}CH_3)_2(O_2C\text{-}CH_3)_2\text{-}(THF)_2$	0	+2	1.26	2.90	11580.378	1.346840	1.15	1.67	2.70	0.10	2.71	EGAFAT
		+2	1.26	2.90	11580.344	1.346839	1.15	2.12	3.43	0.46	3.55	
(28) $Fe_2(\mu\text{-}O_2C\text{-}CH_3)_2(O_2C\text{-}CH_3)_2\text{-}(NH_2CH_2CH_3)_2$	4	+2	1.19	2.90	11580.525	1.346848	1.13	1.72	2.79	0.41	2.86	ADIGID
		+2	1.19	2.90	11580.525	1.346849	1.13	1.73	2.80	0.40	2.88	
(28a) $Fe_2(\mu\text{-}O_2C\text{-}CH_3)_2(O_2C\text{-}CH_3)_2\text{-}(NH_2CH_2CH_3)_2$	0	+2	1.19	2.90	11580.531	1.346848	1.13	1.83	2.95	0.19	2.97	ADIGID
		+2	1.19	2.90	11580.547	1.346854	1.12	2.14	3.46	0.18	3.47	
(29) $Fe_2(\mu\text{-}OH_2)_2(\mu\text{-}O_2C\text{-}CH_3)_2\text{-}(O_2C\text{-}CH_3)_3(THF)_2(OH_2)$	4	+2	1.35	3.26	11579.864	1.346780	1.31	1.70	2.75	0.14	2.76	FEMTEX
		+2	1.35	3.26	11579.769	1.346778	1.31	1.69	2.73	0.46	2.83	
(29a) $Fe_2(\mu\text{-}OH_2)_2(\mu\text{-}O_2C\text{-}CH_3)_2\text{-}(O_2C\text{-}CH_3)_3(THF)_2(OH_2)$	0	+2	1.35	3.26	11579.919	1.346784	1.30	1.87	3.03	0.96	3.46	FEMTEX
		+2	1.35	3.26	11579.766	1.346778	1.31	1.67	2.69	0.45	2.78	
(30) $Fe_2BPMP(OPr)_2^+$	0	+2	1.24	2.72	11579.595	1.346802	1.25	1.41	2.27	0.39	2.33	GATFUC
		+2	1.24	2.72	11579.599	1.346803	1.25	1.41	2.29	0.36	2.34	
(31) $Fe(II)Fe(III)BPMP(OPr)_2^{2+}$	1/2	+2	1.15	2.69	11580.316	1.346865	1.09	1.85	3.00	0.08	3.00	GATFOW
		+3	0.50	0.50	11581.514	1.347034	0.65	0.17	0.27	0.67	0.29	

cont.

(32) $Fe_2(O_2CH)_2(BIPhMe)_2$	0	+2	1.26	2.56	11579.831	1.346800	1.26	1.78	2.87	0.12	2.88	SISKOU
		+2	1.25	3.30	11580.319	1.346840	1.15	2.08	3.36	0.55	3.53	
(33) $Fe_2(OAc)_2(TPA)_2^{2+}$	0	+2	1.12	3.33	11580.138	1.346832	1.17	1.96	3.16	0.68	3.40	VUNMIA
		+2	1.12	3.33	11580.215	1.346844	1.14	1.72	2.79	0.27	2.82	
(34) $Fe_2(ImH)_2(XDK)(O_2CPh)_2(MeOH)$	0	+2	1.35	3.04	11579.625	1.346778	1.31	1.77	2.86	0.59	3.02	YUZKAF10
		+2	1.12	2.83	11580.718	1.346874	1.06	1.31	2.13	0.99	2.45	
(35) $Fe_2(py)_2(O_2CAr^{Mes})_4$	0	+2	1.14	3.23	11580.408	1.346853	1.12	1.52	2.46	0.73	2.67	XIGDIA
		+2	1.14	3.23	11580.409	1.346853	1.12	1.52	2.46	0.73	2.67	
(36) $Fe_2(H_2O)(O_2CPh)_4(TMEN)_2$	0	+2	1.25	3.11	11579.860	1.346793	1.28	1.93	3.12	0.16	3.13	VUPJUL
		+2	1.26	2.70	11579.807	1.346791	1.28	1.83	2.97	0.13	2.98	
(37) $Fe_2(H_2O)(OAc)_4(TMEN)_2$	2	+2	1.27	2.75	11580.012	1.346818	1.21	1.87	3.03	0.03	3.03	VUPJOF
		+2	1.27	2.75	11580.013	1.346817	1.21	2.06	3.32	0.23	3.35	
(38) $Fe(NO)_2(S(p\text{-}Me)Ph)_2^-$	2	+2	0.18	0.69	11582.701	1.347132	0.40	0.46	0.75	0.63	0.79	SONMUE
(39) $[Fe(SC_2H_3N_3)(SC_2H_2N_3)(NO)_2]$	5/2	+3	0.188	1.118	11583.189	1.347218	0.17	0.56	0.91	0.68	0.98	EYABOV
(40) $Fe_2(S\text{-}t\text{-}Bu)_2(NO)_2$	0	+3	0.15	0.90	11583.320	1.347249	0.09	0.36	0.59	0.43	0.61	GIDKIN02
		+3	0.15	0.90	11583.320	1.347249	0.09	0.36	0.59	0.43	0.61	
(41) $Fe(S\text{-}t\text{-}Bu)_3NO$	5/2	+3	0.26	0.46	11583.138	1.347180	0.27	0.11	0.18	0.57	0.19	WEDXAF
(42) $[Fe(NO)(dtc\,i\text{-}Pr_2)_2]$	3/2	+3	0.35	0.89	11582.603	1.347143	0.37	0.24	0.39	0.44	0.41	PRCBFE
(43) $[Fe_2(NO)_2(Et\text{-}HPTB)(O_2CPh)]^{2+}$	0	+3	0.67	1.44	11581.513	1.347036	0.64	0.71	1.15	0.33	1.17	RABHAD
		+3	0.67	1.44	11581.532	1.347039	0.64	0.73	1.19	0.20	1.19	
(44) FeF_6^{4-}	2	+2	1.48	2.85	11579.703	1.346767	1.34	1.71	2.76	0	2.76	ICSD26603
(45) $FeCl_4^{2-}$	2	+2	1.05	3.27	11581.197	1.346893	1.01	1.90	3.08	0.06	3.08	DEBWEM
(46) $FeBr_4^{2-}$	2	+2	1.12	3.23	11581.194	1.346881	1.05	1.99	3.21	0.16	3.22	DEBWIQ
(47) $Fe(NCS)_4^{2-}$	2	+2	0.97	2.83	11581.038	1.346928	0.92	1.76	2.84	0.02	2.84	KEFFEG
(48) $Fe(H_2O)_6^{2+}$	2	+2	1.39	3.38	11579.688	1.346772	1.33	1.82	2.94	0.02	2.94	ICSD16589
(49) $Fe(bipy)_2Cl_2^+$	5/2	+3	0.54	0.24	11582.032	1.347082	0.52	0.31	0.50	0.73	0.55	CAVDOS05
(50) FeF_6^{3-}	5/2	+3	0.61	0.00	11582.155	1.347126	0.41	0.01	0.01	0.91	0.02	TUKBOQ
(51) $FeCl_6^{3-}$	5/2	+3	0.56	0.04	11582.177	1.347069	0.56	0.03	0.04	0.76	0.05	DALLIL
(52) $FeCl_4^-$	5/2	+3	0.36	0.00	11583.208	1.347158	0.33	0.01	0.02	0.42	0.02	MICYFE10
(53) K_2FeO_4	1	+6	-0.90	0.00	11587.208	1.347668	-1.01	0.02	0.03	0.11	0.03	ICSD32756
(54) $FeCl_5(H_2O)^{2-}$	5/2	+3	0.49	0.56	11582.272	1.347090	0.50	0.34	0.55	0.43	0.56	VOCBAQ
(55) $Fe(DTSQ)_2^{2-}$	2	+2	0.67	4.01	11582.068	1.346977	0.80	1.74	2.82	0.94	3.21	PTSQFE10
(56) $Fe(SPh)_4^{2-}$	2	+2	0.66	3.24	11581.983	1.346984	0.78	1.46	2.36	0.85	2.63	PTHPFE10

cont.

(57) $[Fe_2S_2(S_2\text{-o-xyl})_2]^{2-}$	5	+3	0.28	0.36	11583.315	1.347162	0.32	0.13	0.22	0.97	0.25	XLDTSF
		+3	0.28	0.36	11583.315	1.347162	0.32	0.13	0.22	0.97	0.25	
(58) $[Fe_2S_2(OPh\text{-}p\text{-}CH_3)_4]^{2-}$	5	+3	0.37	0.32	11583.059	1.347159	0.32	0.28	0.45	0.18	0.45	GIBCUP
		+3	0.37	0.32	11583.058	1.347159	0.32	0.30	0.48	0.65	0.52	
(59) $[Fe_2S_2(C_4H_4N)_4]^{2-}$	5	+3	0.26	0.49	11582.859	1.347217	0.17	0.39	0.64	0.20	0.64	CONSED10
		+3	0.26	0.49	11582.860	1.347217	0.17	0.39	0.63	0.19	0.64	
(60) $Fe(SEt)_4^-$	5/2	+3	0.25	0.62	11583.239	1.347139	0.37	0.15	0.24	0.07	0.24	CANDAW10
(61) $Fe(PPh_3)_2$(“S2”)$_2$	1	+4	0.16	1.52	11583.052	1.347211	0.19	0.91	1.48	0.15	1.48	SOCVUB
(62) $Fe(PPh_3)$(“S2”)$_2$	0	+4	0.12	3.03	11583.183	1.347192	0.24	1.65	2.67	0.73	2.90	SOCWAI
(63) Fe(OEP)CO	0	+2	0.27	1.84	11582.661	1.347158	0.33	1.03	1.66	0.02	1.66	YEQPOA
(64) Fe(OEP)	1	+2	0.63	1.71	11581.467	1.346999	0.74	1.68	2.72	0.58	2.87	DEDWUE
(65) Fe(OEC)	1	+2	0.62	2.55	11580.971	1.346973	0.81	1.36	2.20	0.84	2.44	BUYKUB10
(66) Fe(OEC)Cl	3/2	+3	0.22	2.99	11583.057	1.347222	0.16	1.40	2.27	0.02	2.27	SUMWUS
(67) $Fe(OEC)C_6H_5$	3/2	+3	-0.08	3.72	11583.786	1.347310	-0.07	1.77	2.85	0.05	2.86	SUMXED
(68) $FeCl(\eta^4\text{-MAC}^*)^-$	5/2	+3	-0.04	0.89	11583.666	1.347301	-0.04	0.42	0.67	0.72	0.73	JESGUJ
(69) $Fe(OEP)(4\text{-}NMe_2Py)_2^{2+}$	1/2	+3	0.26	2.15	11582.271	1.347170	0.30	1.30	2.11	0.04	2.11	VOFLOR

The ligands are encoded as follows: salmp = 2-bis(salicylideneamino)methylphenolate, opda = 1,2-phenylenediamine, BPMP = 2,6-bis(bis(2-pyridylmethyl) aminomethyl))-4-methylphenolato, Me_3TACN = 1,4,7-trimethyl-1,4,7-triazacyclonane, BIPhMe = bis(1-methylamidazol-2-yl)phenylmethoxymethane, $HB(mtda^R)_3$ = tris(mercaptothiadiazolyl)borate, TPA = tris(2-pyridylmethyl)amine, ImH = imidazole, XDK = acid anion of *m*-xylenediamine bis(Kemp’s triacid)-imide, HO_2CAr^{Mes} = 2,6-bis(mesityl)benzoic acid, OEC = dianion of *trans*-7,8-dihydro-octaethylporphyrin, OEP = dianion of octaethylporphyrin, TMEN = N,N,N’,N’-tetramethylethylenediamine, DTSQ = bis(dithiodithiosquarato,S,S’), cy-ac = anion of 1,4,8,11-tetraazacyclotetradecane-1-acetate, cat = catecholato-O,O,O’)-bis(catecholato-O,O’), η^4-MAC* = 13,13-diethyl-2,2,5,5,7,7,10,10-octamethyl-1,4,8,11-tetra-azatetradecan-3,6,9,12,14-pentaone-N,N’,N’’,N’’’, $HBpz_3$ = hydrotis-1-(pyrazolyl)borate, Piv = pivalate, TTC = tetrachlorocatecholato-O,O’ dianion, TMIP = tris(methylimidazol-2-yl)phosphine, MBTHx = bis(*N*-methylbenzothiohydroxamato), $H_2B(MesIm)_2$ = dihydrobis[1-(2,4,6-trimethylphenyl)imidazole-2-ylidene]borato, Et-HPTB = N,N,N’,N’-tetrakis(N-ethyl-2-benzimidazolylmethyl)-1,3,diaminopropane), TAML = tetra-amido macrocyclic ligand, “S2” = 1,2-benzenedithiolato-S,S’ dianion.

a)

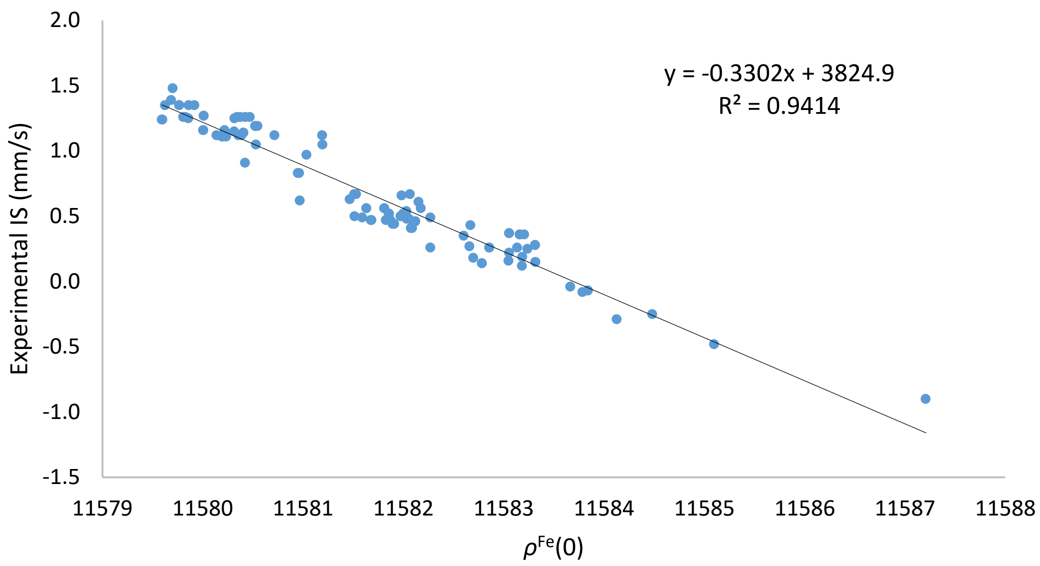

b)

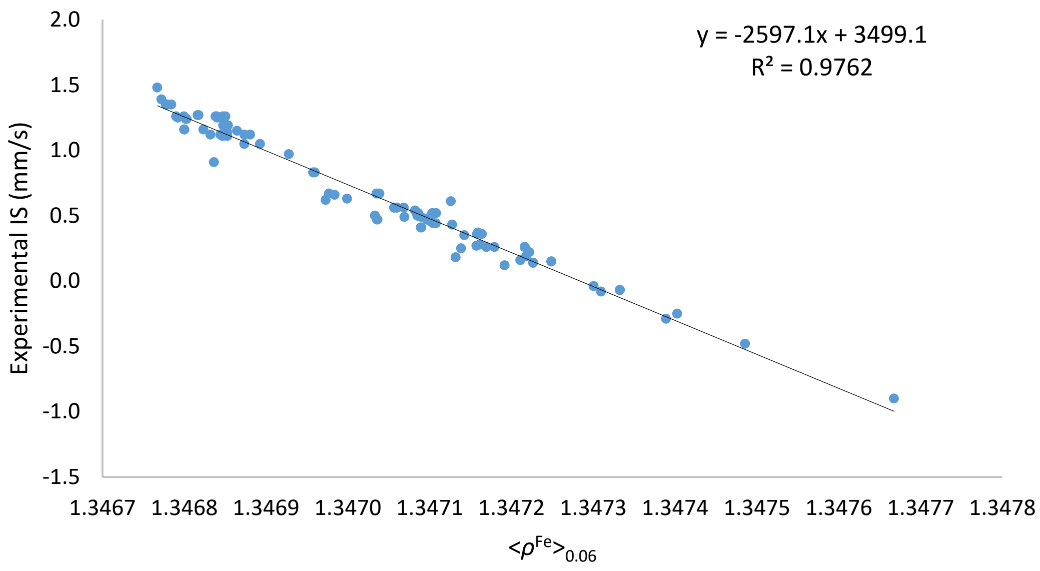

Figure S1: B3LYP calibration lines linear using the value of the electron density (a) at the ^{57}Fe nucleus, (b) integrated in a sphere of radius 0.06 au

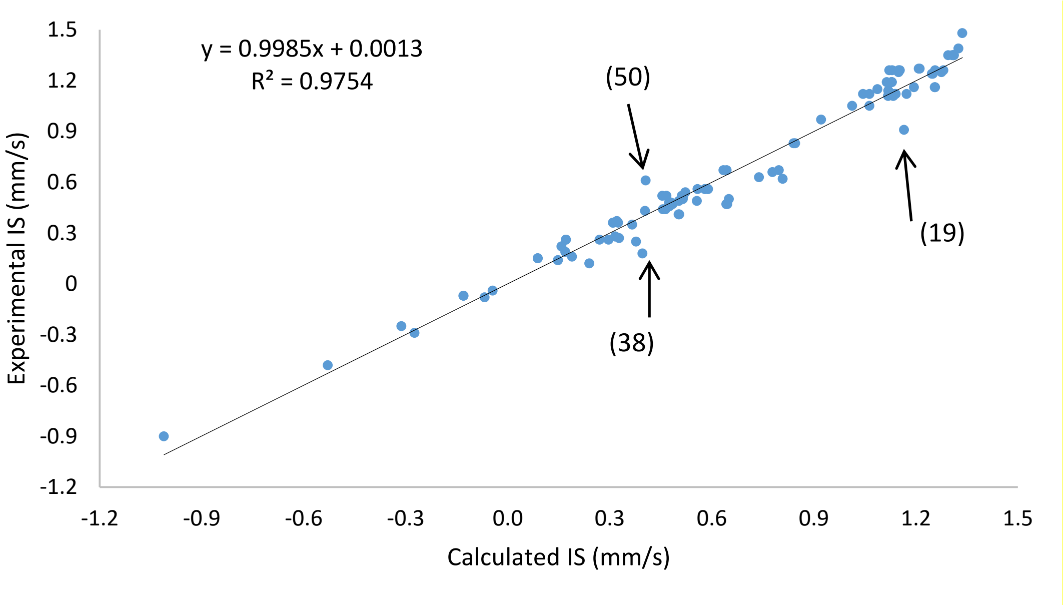

Figure S2: Cross-validation results for the calibration line using $<\rho^{Fe}>_{0.06}$ values (B3LYP).

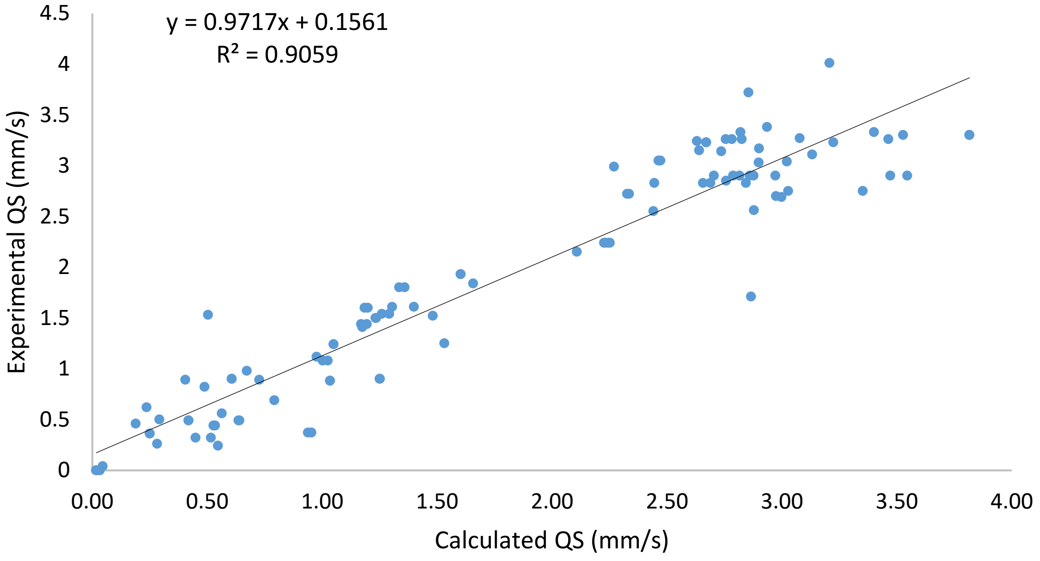

Figure S3: correlation between the experimental and calculated Quadrupole Splitting (B3LYP).

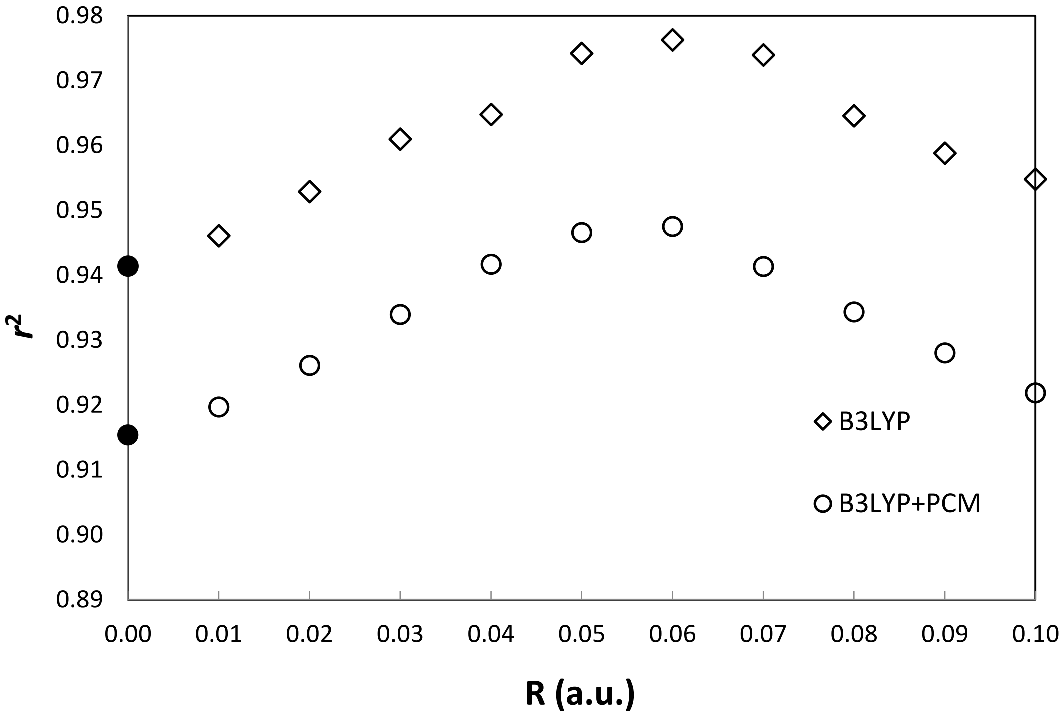

Figure S4: Square of the correlation coefficient of the IS calibration versus the radius of the sphere around Fe. Values at R = 0 correspond to calibration lines calculated using $\rho^{Fe}(0)$.

Table S2: Calculated and Experimental Isomer Shifts and Quadrupole splitting (in mm/s) for the molecular set using $\langle\rho^{Fe}\rangle_{0.06}$ values at PW91/def2tzvp level of theory. Electron densities in atomic units.

			Experimental		Calculated							Code
Complex	S_{total}	OS	$\delta_{4.2K}$	ΔE_Q	ρ(0)	$\langle\rho\rangle_{0.06}$	δ_{calc}	V_{zz}, a.u.	$eQV_{zz}/2$	$\eta_{(calc)}$	$\Delta E_{Q(calc)}$	
(1) $Fe_2(salmp)_2^{2-}$	0	+2	1.11	2.24	11579.796	1.346837	1.05	0.87	1.41	0.21	1.42	KASFUF
		+2	1.11	2.24	11579.833	1.346842	1.03	0.88	1.43	0.24	1.44	
(1a) $Fe_2(salmp)_2^{2-}$	4	+2	1.11	2.24	11579.786	1.346836	1.05	0.89	1.44	0.45	1.48	KASFUF
		+2	1.11	2.24	11579.823	1.346841	1.04	0.88	1.43	0.42	1.47	
(2) $Fe_2(OH)(OAc)_2(Me_3TACN)_2^{+}$	0	+2	1.16	2.83	11579.454	1.346768	1.26	1.56	2.53	0.04	2.53	DIBWUG10
		+2	1.16	2.83	11579.457	1.346768	1.26	1.54	2.50	0.08	2.50	
(3) $Fe_2(salmp)_2^{-}$	9/2	+2.5	0.83	1.08	11580.106	1.346884	0.91	0.38	0.62	0.83	0.69	KASGAM
		+2.5	0.83	1.08	11580.102	1.346884	0.91	0.39	0.63	0.81	0.70	
(4) $Cl_3FeOFeCl_3^{2-}$	0	+3	0.36	1.24	11582.125	1.347062	0.36	0.57	0.92	0.04	0.92	FACTEI
		+3	0.36	1.24	11582.124	1.347062	0.36	0.57	0.92	0.06	0.92	
(5) $Fe_2O(OAc)_2(Me_3TACN)_2^{2+}$	0	+3	0.47	1.50	11580.590	1.346929	0.77	0.58	0.93	0.73	1.01	DIBXAN10
		+3	0.47	1.50	11580.586	1.346928	0.77	0.58	0.94	0.73	1.02	
(6) $Fe_2O(OAc)_2(bipy)_2Cl_2$	0	+3	0.41	1.80	11580.997	1.346982	0.61	0.63	1.02	0.77	1.11	VABMUG
		+3	0.41	1.80	11581.014	1.346983	0.60	0.61	0.98	0.75	1.07	
(7) $Fe_2(salmp)_2$	0	+3	0.56	0.88	11580.540	1.346951	0.70	0.63	1.02	0.77	1.11	KASFOZ
		+3	0.56	0.88	11580.542	1.346951	0.70	0.61	0.98	0.75	1.07	
(8) $Fe_2(cat)_4(H_2O)_2^{2-}$	0	+3	0.56	0.90	11580.723	1.346947	0.72	0.67	1.08	0.82	1.19	TEMKUR
		+3	0.56	0.90	11580.724	1.346947	0.72	0.66	1.07	0.82	1.19	
(9) $Fe_2(O)_2(6\text{-}Me_3\text{-}TPA)_2^{2+}$	0	+3	0.50	1.93	11580.966	1.346990	0.58	0.87	1.40	0.75	1.53	YOCKAC
		+3	0.50	1.93	11580.966	1.346990	0.58	0.87	1.40	0.75	1.53	
(10) $(Fe(Me_3TACN)(TTC))_2O$	0	+3	0.46	1.41	11581.084	1.346999	0.56	0.70	1.13	0.65	1.20	YOHMOX
		+3	0.46	1.41	11581.084	1.346999	0.56	0.70	1.13	0.65	1.20	
(11) $Fe_2O_2(5\text{-}Et_3\text{-}TPA)_2^{3+}$	9/2	+3.5	0.14	0.49	11581.728	1.347125	0.17	0.30	0.49	0.13	0.49	DEKNOW
		+3.5	0.14	0.49	11581.728	1.347125	0.17	0.42	0.68	0.06	0.68	
(12) $(Fe(TAML)_2)_2O^{2-}$	0	+4	-0.07	3.30	11582.699	1.347221	-0.12	1.86	3.00	0.18	3.02	KAJBIH
		+4	-0.07	3.30	11582.699	1.347221	-0.12	1.86	3.00	0.18	3.02	
(13) $Fe_2(OH)(O_2P(OPh)_2)_3(HBpz_3)_2^{2+}$	1	+3	0.44	0.44	11580.994	1.347025	0.48	0.31	0.49	0.32	0.50	PIMTAG
		+3	0.44	0.44	11580.985	1.347022	0.49	0.31	0.50	0.33	0.51	

cont.

(14) $Fe_2O(Piv)_2(Me_3TACN)_2^{2+}$	0	+3	0.48	1.54	11580.916	1.346986	0.60	0.75	1.22	0.52	1.27	ZOCPEM
		+3	0.48	1.54	11580.948	1.346989	0.59	0.75	1.21	0.54	1.26	
(15) $Fe_2O(TMIP)_2(OAc)_2^{2+}$	0	+3	0.52	1.61	11580.915	1.346996	0.56	0.73	1.18	0.78	1.29	JIGNUI
		+3	0.52	1.61	11580.938	1.347001	0.55	0.69	1.12	0.73	1.22	
(16) $Fe_2O(HBpz_3)_2(OAc)_2$	0	+3	0.52	1.60	11580.811	1.346985	0.60	0.70	1.12	0.59	1.19	CACZIP10
		+3	0.52	1.60	11580.810	1.346985	0.60	0.71	1.15	0.53	1.21	
(17) $Fe_2OH(HBpz_3)_2(OAc)_2$	0	+3	0.47	0.37	11580.843	1.347001	0.55	0.58	0.94	0.48	0.98	COCJIN
		+3	0.47	0.37	11580.798	1.346997	0.56	0.56	0.91	0.56	0.96	
(18) $Fe(phen)_2Cl_2$	2	+2	1.05	3.15	11580.178	1.346871	0.94	1.01	1.64	0.20	1.65	CPENFE01
(19) $Fe(opda)_2Cl_2$	2	+2	0.91	3.17	11579.860	1.346802	1.16	1.60	2.59	0.25	2.62	FUJQOQ
(20) $Fe(Py)_4Cl_2$	2	+2	1.16	3.14	11579.767	1.346806	1.14	1.38	2.22	0.01	2.22	TPYFEC
(21) $Fe(HB(mtda^R)_3)_2$	0	+2	0.49	0.26	11580.996	1.347037	0.44	0.12	0.19	0.67	0.20	JOHCEP
(22) $[(Me_3cy\text{-}ac)FeN]^{2+}$	0	+2	-0.29	1.53	11582.915	1.347267	-0.26	0.25	0.41	0.97	0.47	Ref 1
(23) $FeCl(MBTHx)_2$	5/2	+3	0.43	0.98	11581.594	1.347012	0.51	0.47	0.75	0.51	0.78	CELVEU
(24) $H_2B(MesIm)_2Fe(NMes)_2$	3/2	+3	-0.25	0.82	11583.500	1.347308	-0.39	0.46	0.74	0.68	0.79	ZACWUZ
(25) $[H_2B(MesIm)_2Fe(NMes)_2]^+$	0	+4	-0.48	1.25	11584.006	1.347379	-0.61	0.70	1.13	0.33	1.15	ZACXAG
(26) $Fe_2(\mu\text{-}O_2C\text{-}CH_3)_4(C_5H_5N)_2$	0	+2	1.12	3.05	11579.919	1.346827	1.08	1.37	2.21	0.53	2.31	EGAFUN
		+2	1.12	3.05	11579.886	1.346824	1.09	1.39	2.25	0.49	2.34	
(27) $Fe_2(\mu\text{-}O_2C\text{-}CH_3)_2(O_2C\text{-}CH_3)_2\text{-}(THF)_2$	4	+2	1.26	2.90	11579.979	1.346824	1.08	1.46	2.35	0.06	2.36	EGAFAT
		+2	1.26	2.90	11579.939	1.346822	1.09	1.35	2.18	0.25	2.20	
(27a) $Fe_2(\mu\text{-}O_2C\text{-}CH_3)_2(O_2C\text{-}CH_3)_2\text{-}(THF)_2$	0	+2	1.26	2.90	11579.865	1.346811	1.12	1.42	2.30	0.06	2.30	EGAFAT
		+2	1.26	2.90	11579.931	1.346821	1.09	1.37	2.22	0.08	2.22	
(28) $Fe_2(\mu\text{-}O_2C\text{-}CH_3)_2(O_2C\text{-}CH_3)_2\text{-}(NH_2CH_2CH_3)_2$	4	+2	1.19	2.90	11580.002	1.346817	1.11	1.40	2.26	0.42	2.33	ADIGID
		+2	1.19	2.90	11580.009	1.346819	1.10	1.41	2.28	0.42	2.34	
(28a) $Fe_2(\mu\text{-}O_2C\text{-}CH_3)_2(O_2C\text{-}CH_3)_2\text{-}(NH_2CH_2CH_3)_2$	0	+2	1.19	2.90	11580.005	1.346816	1.11	1.65	2.66	0.09	2.67	ADIGID
		+2	1.19	2.90	11580.006	1.346816	1.11	1.66	2.68	0.09	2.68	
(29) $Fe_2(\mu\text{-}OH_2)_2(\mu\text{-}O_2C\text{-}CH_3)_2\text{-}(O_2C\text{-}CH_3)_3(THF)_2(OH_2)$	4	+2	1.35	3.26	11579.256	1.346739	1.35	1.57	2.54	0.19	2.56	FEMTEX
		+2	1.35	3.26	11579.256	1.346751	1.31	1.54	2.49	0.51	2.59	
(29a) $Fe_2(\mu\text{-}OH_2)_2(\mu\text{-}O_2C\text{-}CH_3)_2\text{-}(O_2C\text{-}CH_3)_3(THF)_2(OH_2)$	0	+2	1.35	3.26	11579.298	1.346743	1.33	1.57	2.53	0.19	2.55	FEMTEX
		+2	1.35	3.26	11579.224	1.346746	1.32	1.54	2.50	0.49	2.59	
(30) $Fe_2BPMP(OPr)_2^+$	0	+2	1.24	2.72	11579.595	1.346802	1.15	1.41	2.27	0.39	2.33	GATFUC
		+2	1.24	2.72	11579.599	1.346803	1.15	1.41	2.29	0.36	2.34	
(31) $Fe(II)Fe(III)BPMP(OPr)_2^{2+}$	1/2	+2	1.15	2.69	11579.939	1.346859	0.98	1.09	1.77	0.37	1.81	GATFOW
		+3	0.50	0.50	11580.251	1.346901	0.86	0.20	0.32	0.59	0.33	

cont.

(32) $Fe_2(O_2CH)_2(BIPhMe)_2$	0	+2	1.26	2.56	11579.309	1.346773	1.24	1.64	2.66	0.13	2.66	SISKOU
		+2	1.25	3.30	11579.791	1.346811	1.12	1.92	3.10	0.48	3.22	
(33) $Fe_2(OAc)_2(TPA)_2^{2+}$	0	+2	1.12	3.33	11579.797	1.346832	1.06	1.43	2.32	0.30	2.35	VUNMIA
		+2	1.12	3.33	11579.797	1.346832	1.06	1.43	2.32	0.30	2.35	
(34) $Fe_2(ImH)_2(XDK)(O_2CPh)_2(MeOH)$	0	+2	1.35	3.04	11579.113	1.346754	1.30	1.56	2.52	0.66	2.69	YUZKAF10
		+2	1.12	2.83	11580.195	1.346843	1.03	1.25	2.02	0.95	2.30	
(35) $Fe_2(py)_2(O_2CAr^{Mes})_4$	0	+2	1.14	3.23	11580.026	1.346844	1.02	1.40	2.27	0.54	2.37	XIGDIA
		+2	1.14	3.23	11580.027	1.346845	1.02	1.40	2.27	0.54	2.37	
(36) $Fe_2(H_2O)(O_2CPh)_4(TMEN)_2$	0	+2	1.25	3.11	11579.322	1.346763	1.28	1.78	2.88	0.32	2.93	VUPJUL
		+2	1.26	2.70	11579.250	1.346758	1.29	1.72	2.79	0.18	2.80	
(37) $Fe_2(H_2O)(OAc)_4(TMEN)_2$	2	+2	1.27	2.75	11579.360	1.346771	1.25	1.55	2.51	0.14	2.52	VUPJOF
		+2	1.27	2.75	11579.428	1.346778	1.23	1.41	2.27	0.30	2.31	
(38) $Fe(NO)_2(S(p\text{-}Me)Ph)_2^-$	2	+2	0.18	0.69	11581.988	1.347076	0.32	0.26	0.41	0.82	0.46	SONMUE
(39) $[Fe(SC_2H_3N_3)(SC_2H_2N_3)(NO)_2]$	5/2	+3	0.188	1.118	11582.402	1.347154	0.08	0.34	0.55	0.59	0.58	EYABOV
(40) $Fe_2(S\text{-}t\text{-}Bu)_2(NO)_2$	0	+3	0.15	0.90	11582.549	1.347189	-0.03	0.21	0.34	0.26	0.34	GIDKIN02
		+3	0.15	0.90	11582.549	1.347189	-0.03	0.19	0.31	0.50	0.32	
(41) $Fe(S\text{-}t\text{-}Bu)_3NO$	5/2	+3	0.26	0.46	11582.176	1.347093	0.27	0.64	1.03	0.24	1.04	WEDXAF
(42) $[Fe(NO)(dtc\,i\text{-}Pr_2)_2]$	3/2	+3	0.35	0.89	11581.821	1.347079	0.31	0.32	0.51	0.42	0.53	PRCBFE
(43) $[Fe_2(NO)_2(Et\text{-}HPTB)(O_2CPh)]^{2+}$	0	+3	0.67	1.44	11580.627	1.346959	0.68	0.69	1.11	0.93	1.27	RABHAD
		+3	0.67	1.44	11580.637	1.346961	0.67	0.66	1.06	0.90	1.20	
(44) FeF_6^{4-}	2	+2	1.48	2.85	11579.520	1.346784	1.20	1.38	2.24	0.13	2.24	ICSD26603
(45) $FeCl_4^{2-}$	2	+2	1.05	3.27	11580.592	1.346851	1.00	1.76	2.85	0.04	2.85	DEBWEM
(46) $FeBr_4^{2-}$	2	+2	1.12	3.23	11580.531	1.346832	1.06	1.83	2.95	0.10	2.96	DEBWIQ
(47) $Fe(NCS)_4^{2-}$	2	+2	0.97	2.83	11580.502	1.346899	0.86	1.50	2.43	0.01	2.43	KEFFEG
(48) $Fe(H_2O)_6^{2+}$	2	+2	1.39	3.38	11579.139	1.346741	1.34	1.72	2.78	0.04	2.79	ICSD16589
(49) $Fe(bipy)_2Cl_2^+$	5/2	+3	0.54	0.24	11581.002	1.346984	0.60	0.28	0.45	0.74	0.49	CAVDOS05
(50) FeF_6^{3-}	5/2	+3	0.61	0.00	11581.185	1.347029	0.46	0.01	0.01	0.89	0.01	TUKBOQ
(51) $FeCl_6^{3-}$	5/2	+3	0.56	0.04	11581.176	1.346972	0.64	0.02	0.03	0.77	0.04	DALLIL
(52) $FeCl_4^-$	5/2	+3	0.36	0.00	11582.192	1.347060	0.37	0.01	0.02	0.43	0.02	MICYFE10
(53) K_2FeO_4	1	+6	-0.90	0.00	11585.870	1.347536	-1.11	0.02	0.03	0.19	0.03	ICSD32756
(54) $FeCl_5(H_2O)^{2-}$	5/2	+3	0.49	0.56	11581.549	1.347000	0.55	0.29	0.48	0.41	0.49	VOCBAQ
(55) $Fe(DTSQ)_2^{2-}$	2	+2	0.67	4.01	11581.457	1.346940	0.74	1.24	2.00	0.08	2.00	PTSQFE10
(56) $Fe(SPh)_4^{2-}$	2	+2	0.66	3.24	11581.426	1.346954	0.69	1.19	1.93	0.82	2.13	PTHPFE10

cont.

(57) $[Fe_2S_2(S_2\text{-o-xyl})_2]^{2-}$	5	+3	0.28	0.36	11582.288	1.347067	0.35	0.13	0.21	0.26	0.22	XLDTSF
		+3	0.28	0.36	11582.288	1.347067	0.35	0.13	0.21	0.26	0.22	
(58) $[Fe_2S_2(OPh\text{-}p\text{-}CH_3)_4]^{2-}$	5	+3	0.37	0.32	11582.048	1.347064	0.35	0.18	0.29	0.62	0.31	GIBCUP
		+3	0.37	0.32	11582.048	1.347064	0.35	0.18	0.29	0.62	0.31	
(59) $[Fe_2S_2(C_4H_4N)_4]^{2-}$	5	+3	0.26	0.49	11581.930	1.347123	0.17	0.40	0.65	0.60	0.69	CONSED10
		+3	0.26	0.49	11581.931	1.347123	0.17	0.40	0.65	0.60	0.69	
(60) $Fe(SEt)_4^-$	5/2	+3	0.25	0.62	11582.206	1.347042	0.43	0.22	0.35	0.05	0.35	CANDAW10
(61) $Fe(PPh_3)_2$(“S2”)$_2$	1	+4	0.16	1.52	11582.017	1.347112	0.21	0.65	1.05	0.32	1.07	SOCVUB
(62) $Fe(PPh_3)$(“S2”)$_2$	0	+4	0.12	3.03	11582.471	1.347133	0.15	1.47	2.38	0.22	2.40	SOCWAI
(63) Fe(OEP)CO	0	+2	0.27	1.84	11581.932	1.347096	0.26	1.10	1.78	0.02	1.78	YEQPOA
(64) Fe(OEP)	1	+2	0.63	1.71	11580.934	1.346966	0.66	0.83	1.35	0.40	1.38	DEDWUE
(65) Fe(OEC)	1	+2	0.62	2.55	11580.971	1.346973	0.63	1.72	2.78	0.88	3.12	BUYKUB10
(66) Fe(OEC)Cl	3/2	+3	0.22	2.99	11582.147	1.347143	0.11	1.58	2.56	0.04	2.56	SUMWUS
(67) $Fe(OEC)C_6H_5$	3/2	+3	-0.08	3.72	11582.721	1.347207	-0.08	1.73	2.80	0.08	2.80	SUMXED
(68) $FeCl(\eta^4\text{-MAC}^*)^-$	5/2	+3	-0.04	0.89	11582.454	1.347177	0.01	0.38	0.61	0.86	0.69	JESGUJ
(69) $Fe(OEP)(4\text{-NMe}_2Py)_2^{2+}$	1/2	+3	0.26	2.15	11581.643	1.347131	0.15	1.07	1.73	0.43	1.78	VOFLOR

The ligands are encoded as follows: salmp = 2-bis(salicylideneamino)methylphenolate, opda = 1,2-phenylenediamine, BPMP = 2,6-bis(bis(2-pyridylmethyl) aminomethyl))-4-methylphenolato, Me_3TACN = 1,4,7-trimethyl-1,4,7-triazacyclonane, BIPhMe = bis(1-methylamidazol-2-yl)phenylmethoxymethane, HB(mtda^R)$_3$ = tris(mercaptothiadiazolyl)borate, TPA = tris(2-pyridylmethyl)amine, ImH = imidazole, XDK = acid anion of *m*-xylenediamine bis(Kemp’s triacid)-imide, HO_2CAr^{Mes} = 2,6-bis(mesityl)benzoic acid, OEC = dianion of *trans*-7,8-dihydro-octaethylporphyrin, OEP = dianion of octaethylporphyrin, TMEN = N,N,N’,N’-tetramethylethylenediamine, DTSQ = bis(dithiodithiosquarato,S,S’), cy-ac = anion of 1,4,8,11-tetraazacyclotetradecane-1-acetate, cat = catecholato-O,O,O’)-bis(catecholato-O,O’), η^4-MAC* = 13,13-diethyl-2,2,5,5,7,7,10,10-octamethyl-1,4,8,11-tetra-azatetradecan-3,6,9,12,14-pentaone-N,N’,N’’,N’’’, $HBpz_3$ = hydrotis-1-(pyrazolyl)borate, Piv = pivalate, TTC = tetrachlorocatecholato-O,O’ dianion, TMIP = tris(methylimidazol-2-yl)phosphine, MBTHx = bis(*N*-methylbenzothiohydroxamato), $H_2B(MesIm)_2$ = dihydrobis[1-(2,4,6-trimethylphenyl)imidazole-2-ylidene]borato, Et-HPTB = N,N,N’,N’-tetrakis(N-ethyl-2-benzimidazolylmethyl)-1,3,diaminopropane), TAML = tetra-amido macrocyclic ligand, “S2” = 1,2-benzenedithiolato-S,S’ dianion.

a)

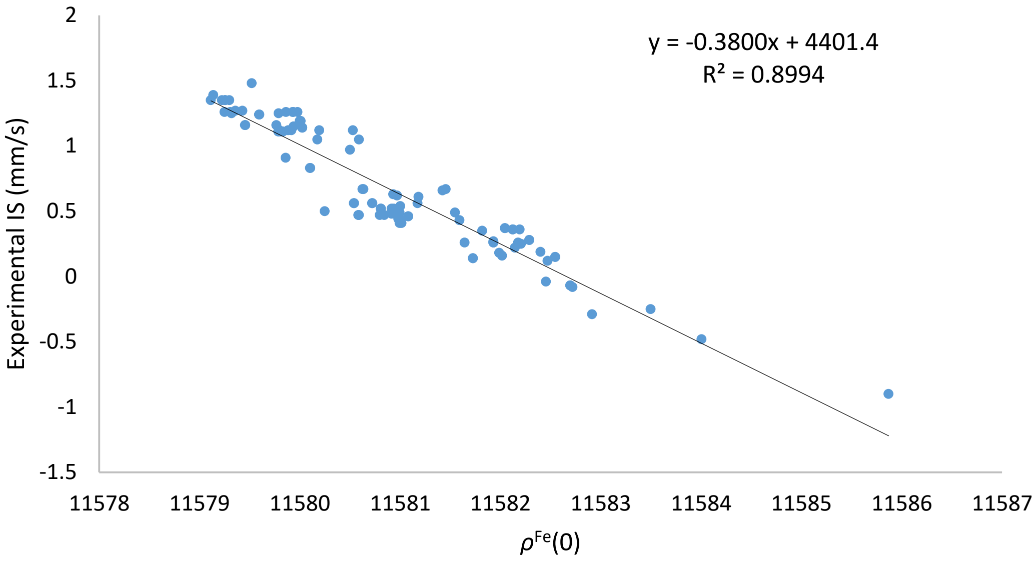

b)

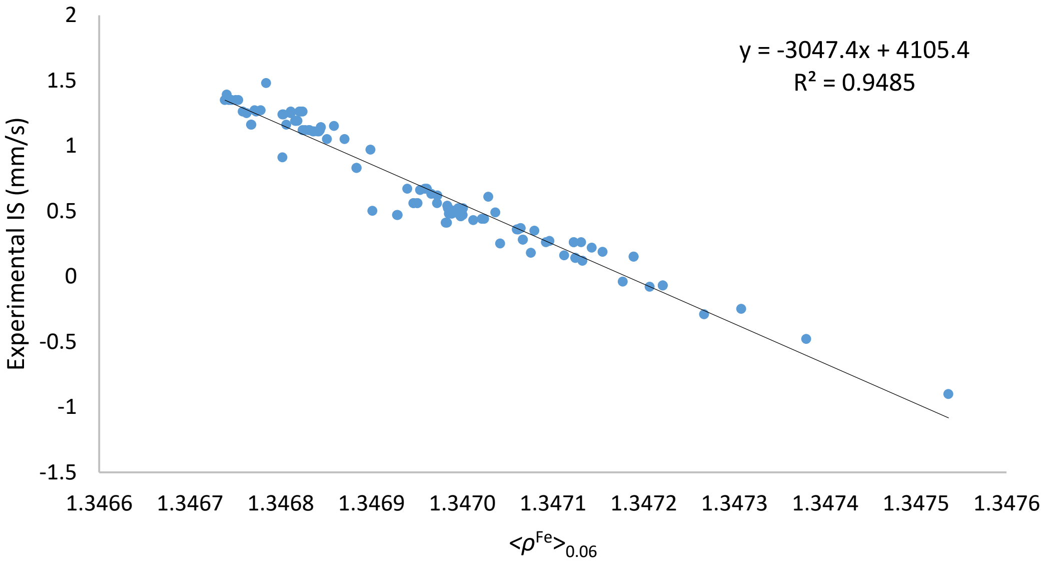

Figure S5: PW91 calibration lines linear using the value of the electron density (a) at the ^{57}Fe nucleus, (b) integrated in a sphere of radius 0.06 au.

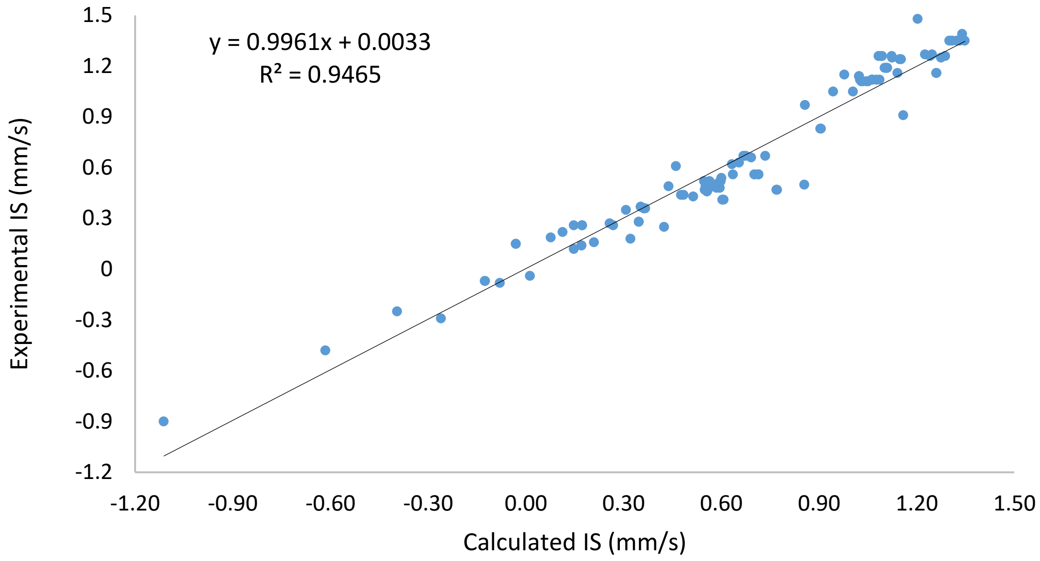

Figure S6: Cross-validation results for the calibration line using <ρ^{Fe}>$_{0.06}$ values (PW91)

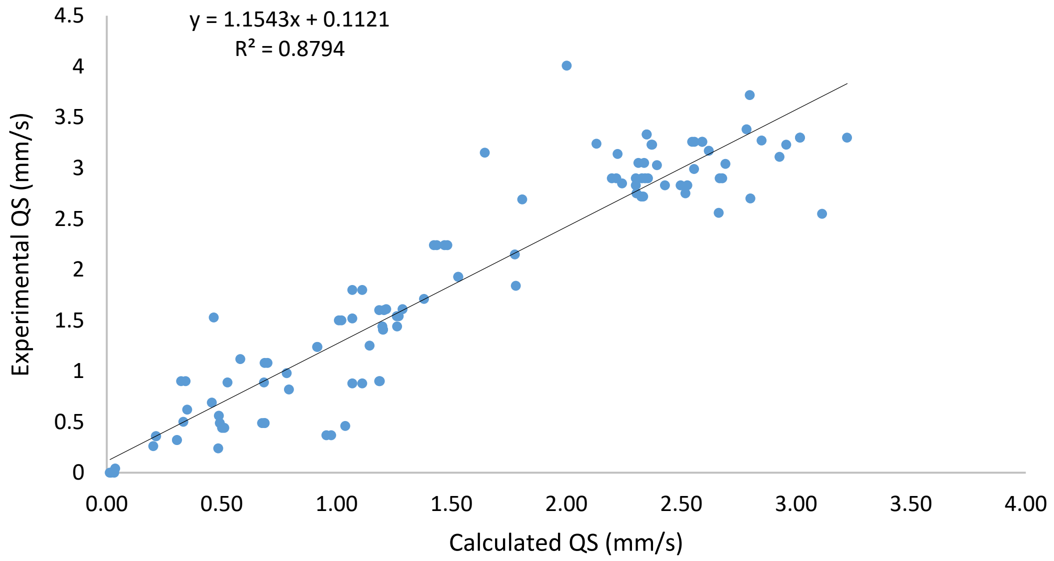

Figure S7: Correlation between the experimental and calculated Quadrupole Splitting (PW91).

Computational Details

All calculations used PW91 and B3LYP functionals in combination with the triple-zeta Gaussian-type basis set def2TZVP. The electron density integrated inside a sphere or radius R centered on the Fe atoms was implemented in our in-house program APOST-3D. For that purpose, a spherical grid of 30 radial and 110 angular points was used. All geometries correspond to x-ray structures except for the high-valent species (22) from Berry et al.[1] The latter corresponds to a BP86/def2svp optimized structure. In some of the high-spin Fe^{+2}/Fe^{+2} diiron compounds (namely, 1, 27, 28 and 29) both the overall ferromagnetic (S=4) and antiferromagnetic states (S=0) are calculated and included in the linear regression, as their relative energy is within 2 kcal/mol. Han et al.[2] also found very low J-coupling values for these compounds. The antiferromagnetic spin state is represented by a "broken-symmetry" (BS) state. Formal oxidation states of the Fe centers in Tables S1 and S2 were obtained using EOS analysis[3] as implemented in APOST-3D code.

References

1. J. F. Berry, E. Bill, E. Bothe, S. D. George, B. Mienert, F. Neese and K. Wieghardt, *Science* **2006**, 312, 1937-1941.
2. W.G. Han, T. Liu, T. Lovell, and L. Noodleman, *J. Comput. Chem*. **2006**, 27, 1292-1306.
3. E. Ramos-Cordoba, V. Postils, P. Salvador, *J. Chem. Theory Comput*. **2015**, 11, 1501-1508
